# Multi-Objective Evolutionary Instance Selection for Regression Tasks

**DOI:** 10.3390/e20100746

**Published:** 2018-09-29

**Authors:** Mirosław Kordos, Krystian Łapa

**Affiliations:** 1 Department of Computer Science and Automatics, University of Bielsko-Biała, ul. Willowa 2, 43-309 Bielsko-Biała, Poland; 2 Institute of Computational Intelligence, Częstochowa University of Technology, 42-201 Częstochowa, Poland

**Keywords:** instance selection, information selection, multi-objective evolutionary algorithms, regression, *k*-NN, computational complexity

## Abstract

The purpose of instance selection is to reduce the data size while preserving as much useful information stored in the data as possible and detecting and removing the erroneous and redundant information. In this work, we analyze instance selection in regression tasks and apply the NSGA-II multi-objective evolutionary algorithm to direct the search for the optimal subset of the training dataset and the *k*-NN algorithm for evaluating the solutions during the selection process. A key advantage of the method is obtaining a pool of solutions situated on the Pareto front, where each of them is the best for certain RMSE-compression balance. We discuss different parameters of the process and their influence on the results and put special efforts to reducing the computational complexity of our approach. The experimental evaluation proves that the proposed method achieves good performance in terms of minimization of prediction error and minimization of dataset size.

## 1. Introduction

Data preprocessing is a crucial step in data mining systems. It is frequently more important than the selection of the best prediction model, as even the best model cannot obtain good results if it learns using poor quality data [1]. A part of data preprocessing is data selection, which comprises feature selection and instance selection.

The purpose of instance selection is to preserve useful information stored in the data and reject the erroneous information, while reducing the data size by selecting an optimal set of instances. This allows for accelerating the predictive model training and to obtain a lower prediction error [2].

Reducing the data size also makes it easier to analyze the properties of the data by humans, as well as allowing the assessment of the expected performance of the prediction models [3]. In other words, by instance selection, we want to “compress the information”. In this work, we consider instance selection in regression problems, which is a more complex task than instance selection in classification problems [4] an much less literature exists on this topic.

Instance selection finds practical application in a range of problems, where the data size can be reduced. For example, it can be applied to the datasets considered in this study, describing real-world problems from various domains. In addition, one of the authors took part in two practical implementations in the industry. The first one was an artificial intelligence-based system for controlling steel production in electric arc process, where there was a lot of data from the previous processes (such as the amount of energy, of different chemical compounds, etc.). The other one was in the electronics industry in a system for predicting the performance of the electronic appliances (power inverters and others) where the amount of data describing the parameters and behavior of the appliances was also very large. In both cases, there were regression problems with many redundant and erroneous data and instance selection was very useful to enable efficient further processing of the data.

**Figure 1:**
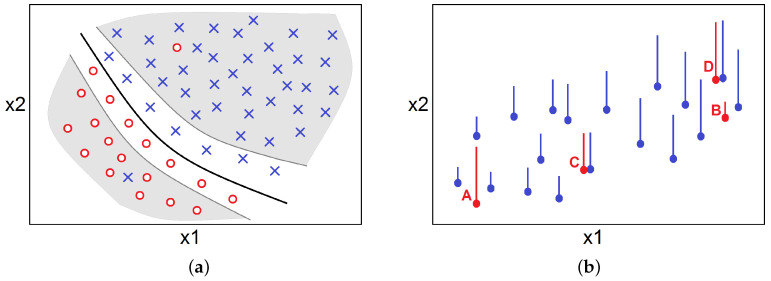
Instance selection in classification (**a**) and in regression (**b**). The axes represent the attributes x1 and x2. In classification, the red circle and blue cross represent points of two different classes. In regression, the height of the vertical line represents the output value of an instance and the circle shows its location in the input space.

The first difference between instance selection for classification and regression tasks that in classification it is enough to determine the class boundaries (the black thick line in Figure 1a), and to select only the instances needed to determine the boundaries [5]. The remaining instances (in the grayed area) can be removed. However, before removing them, the noisy instances, which do not match their neighbor class, must be removed first in order not to introduce the false classification boundaries.

In the case of instance selection for regression tasks, we also need to remove the noisy instances, which do not match their neighbors (instances A and B in Figure 1b) and then we can remove the instances that are very close to some other instances in terms of input and the output space (instances C and D in Figure 1b). However, the reduction cannot be so strong as in classification problems, where we need only the class boundaries because, in regression, each point in the data space is important.

In instance selection for classification tasks, we can obtain strong data reduction and we can also obtain more balanced class distribution and thus higher entropy *H* calculated as [6]:

(1) $$H=-\sum_{i=1}^{c}p\left(X_{i}\right)\log p\left(X_{i}\right),$$

where *c* is the number of classes in the initial dataset and $p(X_{i})$ is the proportion of instances of *i*-th class to all instance in the dataset.

In regression problems, we can estimate the dependent variable *y* as a deterministic continuous function of the independent variables *x* and use differential entropy $H_{d}$ [7]:

(2) $$H_{d}=-\int_{S}f\left(x\right)\log\left(f(x)\right)dx,$$

where *S* is area covered by *x*. We discuss the connections between measures of information and loss functions with instance selection performance in Section 3.4.

However, in practice, instance selection is not so simple as in Figure 1, where there are only two attributes and a few instances. We cannot say which instance needs to be rejected without taking into account which other instances are also rejected. This is because the outcome depends on the set on which the predictive models are trained and thus we must consider the set of selected instances as a whole, which makes instance selection an NP-hard problem (see Section 3.1). For that reason, we decided to base the instance selection method on evolutionary algorithms, where the advantage of this approach is that the evolutionary algorithm evaluates the prediction quality on the entire subsets of selected instances and we do not have to explicitly define the relation of an instance to their neighbors (which in regression tasks is not as simple as in classification) to decide upon its selection or rejection.

Another advantage of evolutionary-based methods is the possibility of obtaining solutions with low prediction error (which in case of regression problems is typically expressed by RMSE—root mean square error) and strong data reduction (compression) at the same time.

As the discussed solution is based on multi-objective optimization, a key advantage of it is that we obtain a pool of solutions situated on the Pareto front, where each of them is the best for certain RMSE-compression balance and we can choose one of them, as will be discussed in Section 2.2.

In binary instance selection, each vector (instance) can be either selected or rejected. In instance weighting, the instances can be assigned real value weights between 0 and 1, which reflect the instance importance for building the predictive model. Then, the model includes the contribution of particular instances in the learning process proportionally to the weights assigned to them [8]. Obviously, we always want to select the most representative instances, so that the reduced set contains as much useful information and as low noise as possible.

First, we review the problems and existing solutions in instance selection with non-evolutionary (Section 1.1) and evolutionary approaches (Section 1.2). Then, we introduce our approach, which uses the multi-objective evolutionary NSGA-II algorithm [9] with *k*-NN as the inner evaluation algorithm and propose several modifications and improvements (Section 2). Finally, the discussion is supported with experimental evaluations (Section 3).

### 1.1. Non-Evolutionary Instance Selection Algorithms

Non-evolutionary instance selection algorithms usually are based on some local properties of the dataset, as the nearest neighbors or Voronoi cells, in order to assess which instances can be removed as noisy or redundant.

The CNN (Condensed Nearest Neighbor) algorithm was the first instance selection algorithm developed by Hart [10]. The purpose of CNN is to reject these instances that do not bring any additional information into the classification process. A popular noise filter is ENN (Edited Nearest Neighbor) proposed by Wilson [11] to increase classification accuracy by noise reduction. These two algorithms are still among the most useful due to their simplicity. In later years, more instance selection algorithms have been proposed for classification tasks [5,12,13,14,15,16,17,18].

In the literature, DROP-3 and DROP-5 are frequently considered the most effective of them [11].

There were also some works to directly use information theory for instance selection in classification tasks. Son [19] proposed a method, where the data set was segmented into several partitions. Then, each partition was divided continuously based on entropy, until all partitions are pure or no further partitioning can be done. Then one can search for the representative instance in each partition. Kajdanowicz [20] introduced a method for comparison and selection of the training set using entropy-based distance.

Recently, adaptations of instance selection methods for multi-output classification problems were proposed [21]. A taxonomy and comparative study of instance selection methods for classification can be found in [2,22].

It is obvious that fewer papers addressed the problem of instance selection in regression tasks and one of the first approaches was presented by Zhang [23]. Since there are no classes in regression, several approaches to replace the “class” with another concept were used.

In [24,25], the class concept was replaced by some threshold distance. If the distance in the input space between two instances is greater than the threshold, they can be treated by the instance selection algorithm in the same way as different class instances in classification tasks. Another option is to perform discretization and convert the regression task to a multiple class classification task and then use instance selection algorithms for classification problems [26]. In [27], a Class Conditional Instance Selection for Regression (CCISR) was proposed, which was derived from the CCIS for classification [28]. CCIS created two graphs: one for the nearest neighbors of the same class as a given instance and another one for other class instances. A scoring function based on the distances in graphs was applied to evaluate the instances.

Guillen et al. [29] proposed the use of mutual information for instance selection in time series prediction. In the first step, the nearest neighbors of a given point were determined and then the mutual information between that point and each of its neighbors was calculated. If the loss of mutual information with respect to its neighbors was similar to the instances near the examined instance, this instance was included in the selected dataset. The authors of [30,31] extended this idea to instance selection in time series prediction by calculating the mutual information between every instance from the training set and the currently evaluated instance, then to order the training set in descending order by the distances and selected the predefined number of points.

In [32], an instance selection method for regression was based on recursive data partitioning. The algorithm started with partitioning the input space using the k-means clustering. If the ratio of the standard deviation to the mean of the group was less than a threshold, the element closest to the mean of each cluster was marked as a representative. Otherwise, the algorithm continued to split the leaf recursively.

In [25], an adaptation of DROP2 and DROP3 to regression tasks was presented and two solutions were proposed: to compare the accumulated error that occurs when an instance is selected and when it is rejected, and to use the already mentioned concept of the distance threshold. Since both ideas were used to adapt DROP2 and DROP3 to regression, four resultant algorithms were tested. DROP3-RT (Regression-Threshold) definitely worked best of the four methods and thus we use it for comparison in the experiments.

In [4], ensembles of instance selection methods for regression tasks were used. The ensembles consisted of several members of the same instance selection algorithm and by implementing bagging operated on different subsets of the original training set. A final decision was made by voting with a threshold. If an instance obtained more votes than the threshold, it was finally selected. Using a different threshold, a Pareto front of solutions could be obtained, i.e., no solution exists, which can improve both of the objectives more than any solution on the front. Results of this work are also used in the experimental comparison.

The problem with non-evolutionary instance selection algorithms is that in most cases they are based on certain assumptions and observations made by their authors about how the data is typically distributed. For example, the ENN algorithm removes the instances miss-classified by *k*-NN, considering them noisy. This is not always true, as they may also be boundary instances and indeed ENN has the tendency to smooth the class boundaries. There are also other assumptions in other algorithms that are more frequently true than not, but in some cases they are wrong.

### 1.2. Evolutionary Instance Selection Algorithms

Evolutionary instance selection algorithms do not make any assumptions about the dataset properties but verify iteratively large number of different subsets in an intelligent way to minimize the search space. This can result in better solutions or better (lower) Pareto front in the case of multiple solutions. On the other hand, this is usually achieved at the expense of much higher computational cost. For that reason, in this work, we pay special attention to limit the computational cost as far as possible, which is discussed in Section 2.3.

Tolvi [33] used genetic algorithms for outlier detection and variable selection in linear regression models, performing both operations simultaneously. However, he evaluated the model on two very small datasets (35 instances with two features and 21 instances with three features); nevertheless, in his experiments, evolutionary instance selection algorithms outperformed the non-evolutionary ones.

Shuning [34] concluded that genetic algorithm based instance selection for classification works best for low entropy datasets and with higher entropy, there will be less benefit from instance selection. In [35], an algorithm called Cooperative Coevolutionary Instance Selection (CCIS) was presented. The method used two populations evaluated cooperatively. The training set was divided into *n* approximately equal parts and each part was assigned to a sub-population. Each individual of a sub-population encoded a subset of training instances. Every sub-population was evolved using a standard genetic algorithm. The second population consisted of combinations of instance sets.

Tsaia [36] considered jointly instance and feature selection in an evolutionary approach. In addition, in [37], an evolutionary algorithm was presented for instance and feature selection and particular problems were assigned to several populations to handle each one separately and each population was optimizing a part of the problem. Then, the authors tried to join the obtained solutions in an attempt to obtain better results. Czarnowski [38,39] introduced an instance selection method that incorporates several ideas. First clustering was performed on the data and then, within the clusters, the selection was executed by the team of agents. The agents cooperated by sharing a population of solutions and refined the solutions using local search.

We found only two works describing the application of multi-objective evolutionary algorithms to instance selection, both to classification problems and both dated for 2017.

In [40], the MOEA/D algorithm was used in a coevolutionary approach integrating instance selection and generating the hyper-parameters for training an SVM. The two criteria used in that optimization were the reduction of the training set size and the performance with a given set of an SVM’s hyper-parameters. The average results over some classification datasets were provided.

In [41], the authors also considered the over-fitting problem. At each iteration of the genetic algorithm, the training and validation partitions were updated in order to prevent the prototypes from over-fitting a single validation data set. Each time the partitions were updated, all of the solutions in the Pareto set were re-evaluated. However, only the results for 1-NN averaged over several classification problems was reported, similarly, as in the previous work, so it is not possible to compare the results with our method.

## 2. Multi-Objective Evolutionary Instance Selection for Regression (MEISR)

This section introduces our solution to instance selection in regression tasks called MEISR. MEISR uses a multi-objective evolutionary algorithm, which in the current implementation is NSGA-II [9], to direct the search for the optimal reduced training sets and the *k*-NN algorithm to determine the error on the training sets. First, particular aspects are discussed and finally the pseudo-code of the whole algorithm is presented.

### 2.1. Basic Concepts

As it was stated in the introduction, the two objectives of instance selection process are minimization of the number of instances in the reduced training set *S* and minimization of the error obtained on the test set by predictive models trained on the reduced training set *S*. The first objective is known as minimization of retention or maximization of reduction or compression. The second one in our case is expressed with root mean square error (RMSE) because RMSE is the standard and commonly used measure of regressor performance. Because the words “RMSE”, “reduction” and “retention” all begin with “r”, we will denote the various RMSE values on the test set with symbols starting with “r” (r0,r1,r2,r3) and we will denote the first objective with symbols starting with “c” (c0,c1,c2,c3), which will stand for retention or 1-compression:

(3) $$c=retention=1-compression=1=\frac{N_{sel}}{N},$$

where *N* is the number of all instances in the training set T, $N_{sel}$ is the number of selected instances from T, which create the selected set S. We use the standard definitions of root mean square error on the training set ($RMSE_{trn}$) and on the test set ($RMSE_{tst}$):

(4) $$RMSE_{trn}=\sqrt{\frac{1}{N}\Sigma_{i=1}^{N}{\left(y_{predicted\_i}-y_{i}\right)}^{2}},$$

(5) $$RMSE_{tst}=\sqrt{\frac{1}{N_{tst}}\Sigma_{i=1}^{N_{tst}}{\left(y_{predicted\_i}-y_{i}\right)}^{2}},$$

where $y_{predicted\_i}$ is the predicted and $y_{i}$ is the actual value of the output variable for the *i*-th instance (of the training and test set respectively) and where *N* is the number of all instances in the training and $N_{tst}$ in the test set. To prevent over-fitting, either validation set or the other stop criterion can be used, as discussed in Section 2.6.

**Figure 2:**
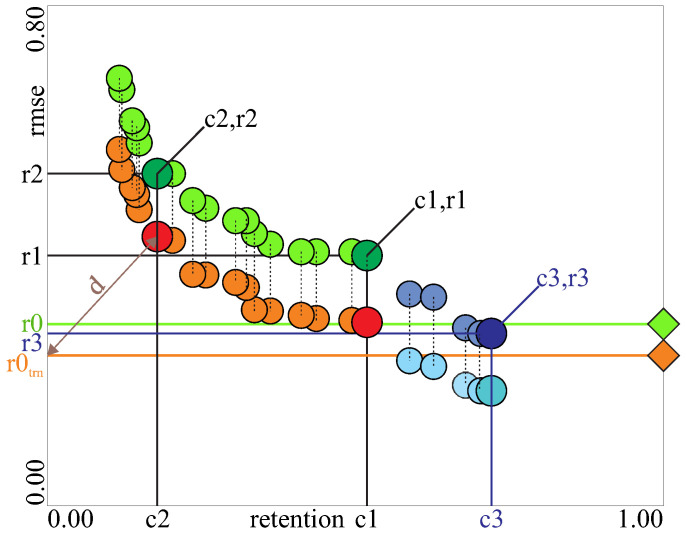
Sample results of the MEISR algorithm run. Each pair of points (orange and green) represent one solution (one training set) with the percentage of selected instances on the horizontal axis and the corresponding $RMSE_{trn}$ on training set (orange) and $RMSE_{tst}$ on test set (green) on vertical axis. Only the points that formed the Pareto front are shown. Horizontal orange and green lines show the $RMSE_{trn}$ and $RMSE_{tst}$ respectively without instance selection. The additional are points shown in blue (c3, r3 and others close to them) are described in the text.

We use a multi-objective evolutionary instance selection method based on the NSGA-II algorithm [9] to maximize compression and minimize RMSE and to obtain a set of solutions on the Pareto front (these are such solutions in which no other solution exists, which can improve both of the objectives more than any solution on the front—see Figure 2).

We chose NSGA-II because it is widely used and, despite the existence of newer algorithms, NSGA-II still gives the best or one of the best results for two-objective problems and its modification NSGA-III for problems with more than two objectives [42]. As the description of NSGA-II can be easily found in the literature [9], we are not going do describe it in detail, but rather discuss the issues specific to the proposed instance selection method, especially that the main focus of our work is on instance selection and not on genetic or evolutionary algorithms.

The NSGA-II algorithm was adjusted to direct the search for solutions for the instance selection task by setting the proper objectives, by using the proper encoding, and by implementing the proposed initialization schemes and mutation and crossover operators, as will be discussed in subsequent sections.

First, an initial population of *P* individuals is created, where each individual represents one reduced training set *S*. The length of the chromosome equals the number of instances in the original training set *T*. At each position of the chromosome, a value *w* represents the weight of a single instance. If *w* = 0, then the instance is rejected. If *w* > 0, then the instance is included in the prediction model (regressor) learning with the weight *w*. In the simplest binary case, we allow only for two different values of *w*: 0 and 1. Initially, all weights *w* have random values generated by the initialization method presented two subsections later.

Then, the evolutionary-based instance selection process starts and by adjusting the weights tries to find a group of the best solutions (reduced training sets *S*), located on the Pareto front. The quality of the solutions during the process is assessed by the two criteria already mentioned: retention (the lower the better) and the prediction error of the learning model (also the lower the better). A detailed discussion of the objective criteria is provided in the next subsection.

### 2.2. The Objectives

The first objective is a minimization of the number of instances of the training set (i.e., minimization of retention or maximization of compression).

The second objective is a minimization of RMSE. It must be distinguished between the final objective, which is a minimization of RMSE on the test set ($RMSE_{tst}$) and the objective used by the instance selection process, which is a minimization of RMSE on the training set ($RMSE_{trn}$). The final objective ($RMSE_{tst}$) cannot be minimized directly because the test set is not available while selecting the instances.

During the instance selection process $RMSE_{trn}$ is internally determined with the leave-one-out procedure, always using the *k*-NN algorithm as the inner prediction model (the regressor inside the instance selection process). In the final evaluation, $RMSE_{tst}$ can be determined using any prediction model trained on the reduced training set *S*. In the experimental section, the final prediction models we used were *k*-NN with different *k* parameters and MLP neural networks and the $RMSE_{tst}$ obtained on the test sets is reported in the experimental results.

Using a single objective genetic algorithm, a fitness function that incorporates all criteria must be defined. The typical definition of the fitness function for instance selection is:

(6) $$fitness={\left(\alpha \cdot retention+\left(1-\alpha \right)\cdot RMSE_{trn}\right)}^{-1},$$

where α is a coefficient indicating the expected balance between the objectives. However, multi-objective solutions do not require the determination of the coefficient α; instead, they minimize two objectives: retention and $RMSE_{trn}$. Moreover, the obtained results consist of a group of non-nominated individuals situated on the Pareto front (i.e., the reduced training sets *S*) with different trade-offs between objectives.

In our study, the following encoding of individuals is used: each individual (each selected data set S) is encoded as a vector $w=\left\{w_{1},\ldots ,w_{N}\right\}$, where *N* stands for the size of the vector (which equals the number of instances in the original training set T). The vector w can only take specific values assigned from set $\left\{0,\sigma ,2\sigma ,3\sigma ,\ldots ,1\right\}$, where σ depends on the algorithm parameter numLevels and it is calculated as follows: $\sigma =1/\left(numLevels-1\right)$. In case of the numLevels=2 vector, w can have only binary values, in case of numLevels=5 vector, w can take five values ($\left\{0.00,0.25,0.50,0.75,1.00\right\}$), etc. If numLevels=0 a vector, w can have any real number values assigned. Such a process was aimed at increasing the readability of weights and the ability to test different variants of simulations.

In the experimental section, we present the results for binary instance selection (numLevels=2) and real-value instance weighting (numLevels=0) as these are two most characteristic encodings. Moreover, implementing instance weighting in regression problems frequently allows for some improvement in prediction quality for noisy data [8,43]. Thus, one of the aims of this work is to examine in what conditions implementing real value weights $w_{i}$ is beneficial, in spite of this making the algorithm more complex and interpretation of the results more difficult.

During the instance selection process, the following objectives are used by NSGA-II for binary instance selection:

(7) $$\begin{array}{c} RMSE_{trn}\left(w\right)=knn\left(S(w),T\right)\\ ret\left(w\right)=\frac{1}{N}\sum_{i=1}^{N}nred\left(w_{i}\right), \end{array}$$

while for real-value instance weighting the following objectives are directly used by NSGA-II:

(8) $$\begin{array}{c} RMSE_{trn}\left(w\right)=knn\left(S(w),T\right)\\ ret\left(w\right)=\beta \cdot \frac{1}{N}\sum_{i=1}^{N}w_{i}+\left(1-\beta \right)\cdot \frac{1}{N}\sum_{i=1}^{N}nred\left(w_{i}\right), \end{array}$$

where $RMSE_{trn}\left(w\right)$ is obtained with the *k*-NN algorithm while predicting output of all instances from the original training set T, using the reduced (selected) training set S given by the weight vector w (each time without the instance currently being predicted). ret(w) in binary instance selection is the sum of instance weights (which equals in this case the number of selected instances), while, in instance weighting, it is a weighted sum of the instance weights $w_{i}$ and the number of instances with non-zero weights $nred(w_{i})$. $nred(w_{i})$ returns 1 if the instance is selected and 0 if it is rejected. β is a parameter that balances the sum of the instance weights and the sum of the not rejected instances. The first term, summing the instance weights, is needed to allow crossover and mutation operations to gradually reduce some of the instance weights $w_{i}$. In case of instance weighting nred(w) is calculated as follows:

(9) $$nred\left(w\right)=\left\{\begin{array}{ccc} 1, & \mathrm{for} & x>\gamma ,\\ 0, & \mathrm{for} & x\leq \gamma , \end{array}\right.$$

where γ is a parameter that defines a weight, below which an instance is rejected (which is experimentally set as 0.01; however, the exact value is not so crucial, as the algorithm adjusts its behavior to that value by modifying the weights proportionally to γ). Such an approach allows the optimization algorithm to minimize the instance weights and, consequently, for a reduction of instances. Instances with weights lower than γ get rejected and are not taken into account by the *k*-NN algorithm, while instances with weights greater than γ are taken into account proportionally to their weights (as well by the inner evaluation as by the final prediction model), as will be discussed in the next section. However, when we report the retention in the experimental results, we take into account the only the number of all instances with non-zero weights.

An example of the obtained Pareto front and the four points of interest are shown in Figure 2. In orange: results on the training set, in green: the corresponding results on the test set. All the points obtained on the Pareto fronts for each dataset are presented numerically and graphically in the supplementary resources. Due to limited space in the paper, we present only four most characteristic points of interest (r0,c0), (r1,c1), (r2,c2) and also (r3,c3) the in cases where r3<r1. Figure 2 is used to explain the results obtained in one fold of the 10-fold cross-validation. The corresponding values reported in the table are the average values over the whole 10-fold cross-validation:

- **r0**—baseline $RMSE_{tst}$ obtained without instance selection.
- **c0**—c0 is always 1, which means there is no compression on the whole dataset, for that reason the value does not occur in any table.
- **r1**—$RMSE_{tst}$ obtained with instance selection for the point (c1,r1) in Figure 2; this is the $RMSE_{tst}$ obtained on the test set, while training the model on this reduced training set *S*, which is the point on the Pareto front with the lowest $RMSE_{trn}$ on the training set and the weakest compression.
- **c1**—retention rate (1-compression) of the point (c1,r1).
- **r2**—$RMSE_{tst}$ obtained with instance selection for the point (c2,r2) in Figure 2; this is the RMSE obtained on the test set, while training the model on this reduced training set, which was represented by the closest obtained point to the point ($r0_{trn}$, retention=0); the brown diagonal line shows this distance *d*. This point was selected as a representative point because further increasing compression usually leads to sudden increase of $RMSE_{trn}$ and $RMSE_{tst}$, making the area to the left of this point practically unusable.
- **c2**—retention rate of the point (c2,r2).
- **r3, c3**—$RMSE_{tst}$ and retention rate obtained with and additional run of the instance selection process with the alternative initialization (90% probability of each instance being included in the initial population). It was aimed to obtain $RMSE_{tst}$ lower than r1 and r0. However, this was useful only in a few cases; in other cases, r3 was equal r0 or to r1, which meant that no further decrease of RMSE below r0 or r1 (whichever was lower) was possible to obtain. One of the important reasons that it was not always possible to obtain the lowest RMSE with the main instance selection process at the point (c1,r1) was that the Pareto front was extending gradually during the optimization and in some cases. Before it would reach the point corresponding to (c3,r3), the test error for the same compression can already start to increase (we minimize $RMSE_{trn}$ and report the $RMSE_{tst}$), so we must stop the process earlier (see Section 2.6).

Although the target users of our method are scientists and engineers, who should understand their process and be able to select the appropriate solution from the Pareto front, we can suggest the solution with the lowest $RMSE_{trn}$ for predictive model learning and the solution marked by the point (c2,r2) in Figure 2 for analyzing the properties of the data. To enable a better choice, it may also be a good idea to display the front graphically (as can be done in the software available in the supplementary resources) so the user can quickly assess all the solutions.

### 2.3. *k*-NN as the Inner Evaluation Algorithm

The rationale behind choosing *k*-NN as the inner evaluation algorithm is the speed of this approach. This is because the full *k*-NN algorithm has to be performed only once before the optimization starts. In the case of other prediction algorithms, this would be either impossible or much more complex and thus less efficient. Let us assume that there are 96 individuals in the population and that the optimization requires 30 epochs. In this case, the value of the fitness function must be calculated 2880 times. Training any prediction model 2880 times would be computationally very costly.

Although, our previous experiments [24] showed that the best results in terms of RMSE-compression balance can usually be obtained if the inner evaluation model is the same algorithm as the final predictor, in this work, we sacrifice that small improvement in order to shorten the optimization process usually from two to three orders of magnitude. However, as we will show, when the final predictor is an MLP neural network, for the inner evaluation, we use *k*-NN with parameters, which makes its prediction as close to the prediction of the neural network as possible. In this way, we obtain better results, while still keeping the process time short.

In the case of the *k*-NN algorithm, we calculate the distance matrix between each pair of instances in the training set. Then, we create one two-dimensional array $D_{i}$ for each instance $X_{i}$. The first dimension is *N*—the number of instances in the training set and the second dimension is three. The three values stored in the array are: $dist(X_{i},X_{j})$, *j* and $y_{j}$. $dist(X_{i},X_{j})$ is the distances between the current instance $X_{i}$ and each other instance $X_{j}$, which in the simplest case is a simple Euclidean distance and in a general case can be expressed by Equation (11). The second value *j* is the ordinal number of each instance in the dataset T. The third value is the output value $y_{j}$ of the *j*-th instance.

Then, we sort the arrays $D_{i}$ increasingly by $dist(X_{i},X_{j})$. At the prediction step, we only go through the beginning of the array, read the instance number *j* and check if this instance is selected, if not, then we go to the next instance, as long as we find *k* selected instances. Then, for the *k* nearest selected instances, we read their weights $w_{j}$ from the chromosome, their output value $y_{j}$ and predict this instance output value $y_{predicted\_i}$ as the weighted average of the *k* outputs (which is a simple average in case of binary instance selection):

(10) $$y_{predicted\_i}=\frac{\sum_{j=1}^{k}y_{j}w_{j}}{k\cdot \sum_{j=1}^{k}w_{j}},$$

where $y_{j}$ is the output value of the *j*-th neighbor of an instance $X_{i}$ and $w_{j}$ is the weight expressing the *j*-th neighbor importance (it should not be confused with the weight $wd_{j}$ related to the distance between the instances $X_{i}$ and $X_{j}$ as in the standard weighted *k*-NN algorithm). Only the neighbors with $w_{j}>\gamma$ are considered. In binary instance selection, $w_{j}=1$ always. To prevent an instance from being considered by the algorithm a neighbor of itself, we set the distance to the instance itself to a very large number as the maximum value of the Double type (1.79 × $10^{308}$). The prediction step is extremely fast and only these steps are performed each time the $RMSE_{trn}$ is calculated.

The distance between two instances $X_{i}$ and $X_{j}$ is calculated as:

(11) $$dist\left(X_{i},X_{j}\right)={\left(\frac{\sum_{m=1}^{A}wa_{m}{\left(x_{im}-x_{jm}\right)}^{v}}{\sum_{m=1}^{A}wa_{m}}\right)}^{1/v},$$

where *A* is the number of features (attributes), $wa_{m}$ is the weight of the *m*-th attribute and $x_{im}$ and $x_{jm}$ are the values of the *m*-th attribute of *i*-th and *j*-th instance respectively. In the simplest case, all the attribute weights equal 1. However, there are cases where it is useful to use attribute weighting and assign different weights to different attributes, e.g., as the absolute values of the correlation coefficient between each attribute and the output. For example, attribute weighting is useful in improving instance selection results if the final predictor is also *k*-NN using the attribute weights or when the final predictor is some other algorithm, which internally performs attribute weighting, e.g., an MLP neural network [44]. *v* is the exponent in Minkovsky distance measure; for v=2, the measure becomes the Euclidean distance. There is usually no need to raise the expression in the bracket to the power 1/v (calculate a square root for v=2) because we need only to find the nearest neighbors and not the distance to them (unless we use weighted *k*-NN, where the closest neighbors have a greater influence on the prediction).

If the final prediction model is i.e., a neural network, then, during the learning, an error the network makes as a response to presenting a given instance is multiplied by the instance weight:

(12) $$Error=\sum_{i=1}^{n}f\left(w_{i}\cdot f\left(y_{predicted\_i}-y_{i}\right)\right),$$

where Error is the total error, used in the network learning algorithms to adjust the network weights and f(.) is an error measure, which, for the frequently used mean square error measure, gives:

(13) $$Error=\sum_{i=1}^{n}w_{i}\cdot {\left(y_{predicted\_i}-y_{i}\right)}^{2}.$$

### 2.4. Population Initialization

The main goal of population initialization methods is to provide optimal coverage of the search space. This can shorten the evolutionary optimization and enable search for the solutions either more uniformly spread in the solution space or focused in some areas, depending on the needs.

Typical initialization assigns randomly generated values (weights) $w_{i}$ to each position *i* at the chromosome (to each instance) of the individuals (training datasets). For this purpose, different methods can be used (e.g., Pseudo-Random Generators [45], Quasi-Random Generators [46], population dependent methods [47]). In addition, methods based on value transformations [48], a priori knowledge about the problem or clustering can be used [49]. According to some authors, initialization should be adjusted to a specified problem [50] and it is also the case in instance selection tasks.

One of the best initialization methods that relies on population is Adaptive Randomness [47]. In this method, for each individual in population several candidates are created. Only the candidate for which that smallest distance to the rest of the population is the larger than for all other candidates is added to the population. The advantage of this method is that the created candidates do not have to be evaluated, only the distance to the already assigned individuals is calculated. Population-based methods do not depend on random number generators and number transformation methods. Due to this, they can be combined with other types of initialization methods [51]. In this work, such combinations have been tested with an idea to produce different initial values (e.g., initialize smaller values and examine the effect of such initialization on the obtained results).

Additionally, several methods that aim at the differentiation of initialized values (to achieve different degrees of instance selection reduction) are introduced:

- Power transformation. In this method, the randomly generated number real number rnd (0<rnd<1) is raised to a certain power *v* as follows:
  (14) $$w_{i}=rnd^{v},$$
  where higher *v* results in lower values (because 0>rnd<1) obtained after transformation.
- Fill transformation. This binary transformation method uses a predefined probability f1 that determines balance between 0 and 1 as a result of initialization:
  (15) $$w_{i}=\left\{\begin{array}{ccc} 0, & \mathrm{for} & rnd<f1,\\ 1, & \mathrm{for} & rnd\geq f1. \end{array}\right.$$
- Spread initialization. The idea behind this method is to differentiate the individuals in population in terms of the different probabilities of occurrence of small and large values:
  (16) $$w_{i}=\left\{\begin{array}{ccc} 0, & \mathrm{for} & rnd<f2,\\ rnd, & \mathrm{for} & rnd\geq f2, \end{array}\right.$$
  where f2=0.1+h∗n/N is a value dependent on particular individual in the population, *n* stands for index of the individual in the population, *h* is a parameter.

In case of binary values, the numbers generated by initialization methods are rounded to the closest acceptable value. Based on the preliminary experiments, which are available in the supplementary resources, we decided to use the adaptive randomness combined with power and fill transformations.

### 2.5. Genetic Operators: Crossover and Mutation

We used the multi-point multi-parent crossover. The number of instances in the original training set *T* (which equals the chromosome length) was *N*. The *M* split points of the chromosome were randomly selected for each child. Then, a parent providing the genetic material for each segment was randomly chosen with the probability proportional to its fitness (thus the same parent could be chosen for more than one segment). The number *M* influences the speed of the algorithm conversion and the experiments showed that the optimal number *M* for the fastest conversion can be roughly expressed as:

(17) $$M=\left\{\begin{array}{ccc} round(n/10) & \mathrm{for} & n\leq 1000,\\ 100+round((n-1000)/100) & \mathrm{for} & n>1000. \end{array}\right.$$

However, smaller numbers *M* than given by Equation (17) also provide similar results in the instance selection, but the optimization takes longer. *P* children were generated in this way. Then, the *P* children and *P* parents were merged together into one group and *P* individuals with the highest fitness value from the merged group were promoted into the next iteration. (It can be said that the probability of crossover was 100% and because of this way of promoting individuals to the next iteration, it was the only optimal probability).

A broad range of mutation probabilities could be used without significantly influencing the results. We used the probability of mutation of 0.1% per each chromosome position and the following mutation operation:

(18) $$w_{i}=\left\{\begin{array}{ccc} w_{i}+\left(rnd-0.5\right)\cdot m_{range} & \mathrm{for} & numLevels=0,\\ RND(0,numLevels-1) & \mathrm{for} & numLevels>0, \end{array}\right.$$

where rnd stands for randomly generated real number from range $\langle 0,1\rangle$, RND(0,numLevels−1) stands for random generated integer number from set {0,…,numLevels−1}, $m_{range}$ stands for mutation range parameter (set experimentally as 0.2).

### 2.6. Training Time Minimization

In genetic algorithms, larger populations require fewer epochs of the algorithm to converge. However, some optimal population size exists from the viewpoint of the computational cost of the whole process. The cost can be approximated by the number of fitness function evaluations. According to our tests, this optimal population size was between 60 and 120 individuals. We decided to use 96 individuals because 96 was a multiple of the number of CPU cores in our server, so it scaled well in parallel computation. Larger populations increase computational costs but do not have any other negative impact on the process. However, if the population would be too small, it may limit the diversity of the individuals and prevent the algorithm from finding the best solutions.

Longer chromosomes, which represent lager datasets, require more epochs of the evolutionary algorithm to find the desired solutions. If too few epochs are used, under-fitting can occur and the solutions can be of poor quality, where the model fits neither the training data nor the test data enough well. On the other hand, using too many epochs can cause over-fitting—the model fits the training data too well and thus fails to fit the test data enough well (does not have generalization capabilities), which prevents good performance on the test data [52,53]. When over-fitting occurs, the error on the training set continuously decreases with further model learning, and the error on the test set starts increasing.

The biggest role in creating over-fitting can be attributed to the model getting fitted to the outstanding, noisy instances, when the model learning runs too long and thus the model gets too complex [52]. In our previous works [44,54], we studied various methods of preventing over-fitting in neural network learning. Noise removal by the ENN instance selection algorithm [11] and its extensions proved to be among the best methods. In the experimental section we compare the MEISR method to the extensions of ENN.

To prevent over-fitting in the MEISR algorithm, we used a standard early stopping approach [53]. By watching the error on a validation test (which is a part of the training set not used for model training but for online evaluation), the process can be stopped before the error starts increasing. In the thousands of experiments, we determined that, for the MEISR algorithm with 96 individuals in the population, the safe number of epochs, which always prevented over-fitting and which was high enough to train the model properly can be expressed as:

(19) $$E=e_{0}\cdot \log\left(N\right),$$

where *N* is the number of instances in the training set T, $e_{0}$ = 8 for binary instance selection and $e_{0}$ = 24 for real value instance weighting. For example, this gives 20 epochs for 300 instances and 34 epochs for 30,000 instances for binary instance selection. Thus, this can be also used as an early stopping criterion, especially that the process proved highly repeatable. This criterion has an additional advantage that it allows using all available instances for the original training set and thus achieving the best possible selected subsets. On the other hand, controlling the error on a validation set can allow for more training epochs and thus better model adjustment. Based on the experiments, we can conclude that the stopping criterion given by Equation (19) more frequently allowed for better results, especially for smaller datasets (where the difference between the validation and test set was high) and also the process was frequently faster.

### 2.7. Pseudo-Code of the MEISR Algorithm

The pseudo-code in Algorithm 1 shows the instance selection process. NSGA-II uses the basic operations of the standard genetic algorithm: crossover and mutation, but, additionally, it utilizes a mechanism to generate a wide Pareto front (the solutions situated on the Pareto front are called non-dominated solutions) by calculating the so called crowding distance [9] between the solutions on the fronts (line 19) and uses it to favor the solutions that are far apart from other ones (line 20).

**Algorithm 1** Multi-objective evolutionary instance selection **Input:** The original data set T **Output:**
F—*F* reduced non-dominated training sets $S_{i}$1:calculate and sort the distance matrices for T2:P:=initialization(N) {population P contains *P* randomly reduced data sets $S_{i}$}3:evaluation(P) {calculate $RMSE_{trn}$ and compression for each $S_{i}$ in P}4:F=fast_nondominated_sort(P,N)5:crowding_distance(F)6:**while** not StopCondition (epoch = E or validation RMSE grows) **do**7: $P^{\prime }=\emptyset$ {new population $P^{\prime }$ will contain children}8: **for** p=1 **to** *P* **do**9:  **for** m=1 **to** *M* **do**10:   parent(m)=select_parent(P)11:   crossover_point(m)=rnd(N)12:  **end for**13:  child(p)=new_individual(parent1,…,parentM)14:  $P^{\prime }=P^{\prime }\cup \mathrm{child}\left(p\right)$15: **end for**16: $\mathrm{evaluation}(P^{\prime })$ {calculate compression and $RMSE_{trn}$ using the distance metrices}17: $P=P\cup P^{\prime }$18: F=fast_nondominated_sort(P,2N)19: crowding_distance(F)20: P=selection(P,F)21:**end while**22:**return**F

## 3. Experimental Evaluation

**Figure 3:**
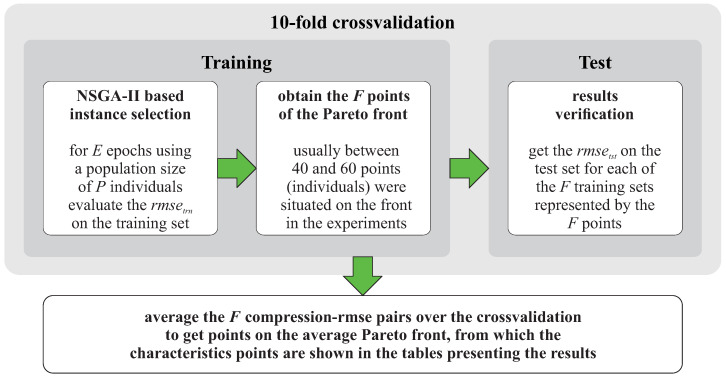
The experimental process.

**Figure 4:**
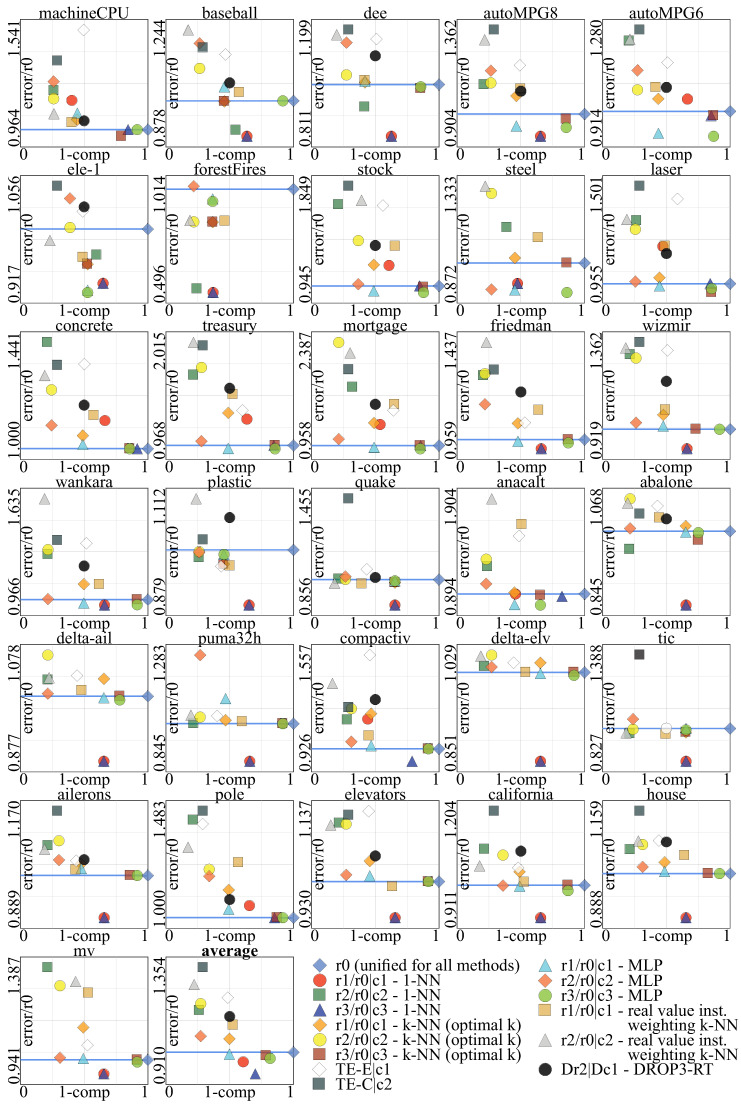
Comparison of the instance selection approaches.

This section presents the experimental evaluation of the MEISR algorithm. The experimental process is presented in Figure 3. To provide comprehensive results, the algorithm is evaluated with prediction models (regressors) that belong to three different groups of models, regarding the response to instance selection (1-NN, *k*-NN, MLP neural network). Finally, a comparison with several other instance selection methods is presented. Most of the analysis is based on the relative $RMSE_{tst}$ (a ratio of RMSE after instance selection to RMSE before instance selection) because our focus here is not to find the best predictive model, but to evaluate how much the performance of the given model will differ after applying instance selection. However, for a reference, we also present the absolute values of RMSE. The dataset properties are presented in Table A1. The experimental results are presented in Table A2, Table A3, Table A4, Table A5, Table A6, Table A7, Table A8, Table A9, Table A10, Table A11, Table A12, Table A13 and Table A14, explained in Figure 2 and its description and summarized in Figure 4.

### 3.1. Data Sets

In case of instance selection, we never know the best solution because it is an NP-hard problem, where trying the number of all possible combinations from sets of thousands of instances is out of accessible computational resources (the number of 5000 combinations from 10,000 instances is about $10^{3010}$ and even the number of 500 combinations from 1000 instances is about $10^{300}$). Thus, we cannot use a dataset with the best results known and see how much we approached this. To ensure reliable and unbiased results, we used the standard benchmark datasets from the KEEL Repository [55], as shown in Table A1. The output values were standardized in the experiments to enable us to evaluate and compare the results better.

### 3.2. Experimental Setup

The experimental setup is shown in Figure 3. We conducted the experiments using our own software written in C# language. Very detailed experimental results and the software source code can be found in the supplementary resources at *kordos.com/entropy2018*. The interested reader can find much more information there than can fit into this paper and replicate the experiments using our software. The experimental process is presented in Figure 3.

Different predictive models display different sensitivity to instance selection. To capture a broad range of model responses to instance selection and at the same time to keep the paper length within a reasonable limit, we carefully chose three models, where each of them is a representative of one model group regarding the sensitivity to instance selection.

The 1-NN algorithm is very sensitive to a change of single neighbor, as the prediction is based on that only neighbor and for this reason it is strongly influenced by instance selection. *k*-NN with higher *k* is less sensitive to a single neighbor change and thus the instance selection influences the $RMSE_{tst}$ to a lower degree than in 1-NN. The distance measures used for the *k*-NN algorithm are described in details in Section 2.3 and the optimal *k* values in Table A1.

There is also a group of predictive models, which base the prediction results (function approximation) on a broad neighborhood and thus their $RMSE_{tst}$ is much less dependent on instance selection; however, instance selection strongly accelerates the learning process. We chose an MLP neural network as a representative of this group, as it is one of the most popular models. We used a network with a typical structure for regression problems: one hidden layer with hyperbolic tangent transfer functions and one neuron in the output layer with the linear transfer function. The hidden layer consisted of six neurons if the number of attributes was below 12 and 12 neurons otherwise and was trained with the VSS algorithm [56] for 15 iterations (as this algorithm does not require more iterations). We also trained it with the well-known Rprop algorithm [57] for 60 epochs and the obtained results were almost the same as for VSS (the differences were statistically insignificant with t-test and Wilcoxon test), thus we do not report them here.

As the best results can be obtained if the evaluation model within instance selection is the same as the final predictive model [24], we used the same models at both positions, with the exception of when the final model was an MLP neural network. In this case, the evaluation model within instance selection was *k*-NN with optimal *k* because of the speed of the solution and the fact that an MLP network response to instance selection is closer to the response of *k*-NN with optimal *k* than to that of 1-NN.

We were able to find in the literature two papers suitable for comparison ([4,25], which have been presented in Section 1.2), which considered instance selection for regression problems and presented the results on a set of several datasets, reporting the obtained compression and RMSE or $r^{2}$ correlation.

In [4], the Pareto front was used. Since the experiments were conducted on most of the same datasets from the Keel Repository using the optimal *k* in *k*-NN, we used the detailed results from the online resources to that paper and performed the comparison. There were eight methods in this paper and we included in comparisons the four best of them, namely: threshold-based CNN ensemble (TE-C), threshold-based ENN ensemble (TE-E), discretization-based CNN ensemble (DE-C), discretization-based ENN ensemble (DE-E) and compared with our MEISR method.

In [25], the results were presented only for a single point with 8-NN for each dataset. We obtained these from the author’s detailed experimental results, which were also performed on most of the same datasets from the Keel Repository. Thus, we conducted the experiments with MEISR using 8-NN for a comparison with their results and measured the output additionally in $r^{2}$ correlation because that measure was used in the DROP3-RT method evaluation. Four methods were presented in this paper and DROP3-RT was the best one, so we included only DROP3-RT in the comparison with MEISR.

All the tests were performed using two testing procedures. The first one was 50% holdout, where randomly chosen 50% of instances were used for training and the remaining instances for testing (in case of odd instance number, the last instance was randomly assigned to one of the sets). The tests were performed 10 times with different random instances chosen for the test and training set each time. The average results over the 10 tests are reported. The reason of repeating this procedure 10 times is based on the recommendations that an experimental design should provide a sufficiently large number of measurements of the algorithm performance and, based on many analyses, 10 experimental measurements is the recommended standard [58,59].

The second procedure was a standard 10-fold cross-validation [59]. In this case, the dataset was first randomly divided into 10 parts with an equal number of instances (or almost equal it the instances cannot be divided equally into 10 parts). Then, the 10 measurements were performed; each of the 10 times a different part of the data was selected as a test set and the remaining nine parts as a training set.

In both cases, we reported the final goals of instance selection: the average RMSE over the 10 test sets and average retention over the 10 training sets.

The two most widely used statistical tests for determining if the difference between the results of two models over various datasets is non-random are the paired *t*-test and Wilcoxon signed-ranks test. The *t*-test assumes that the differences between the two compared random variables are distributed normally and is also affected by outliers which may decrease its power by increasing the estimated standard error. The Wilcoxon signed-ranks test does not assume normal distributions and is less influenced by outliers. However, when the assumptions of the paired *t*-test are valid, it is more powerful than the Wilcoxon test [60]. We used both of them and, in each case, both of them equally indicated the significance of the difference for the standard *p*-value of 0.05.

The standard deviations of the RMSE and retention in the experiments can be found in the online supplementary resources. We do not report them here to save the space because they are not used by the statistical tests, either by any other analysis.

### 3.3. Experimental Results and Discussion

The experimental results are summarized in Figure 4 (the figure presents the results in 10-fold cross-validation, but the results in 50% holdout were very similar). The numerical results for both testing procedures 50% holdout and 10-fold cross-validation are placed in the tables, where Table A2 summarizes all the results. The reduction (compression) is obviously independent of the final prediction model because the model is not used during the instance selection. However, RMSEtst depends on the final prediction model.

The average influence of instance selection on the results in both testing procedures: 50% holdout and 10-fold cross-validation was very similar, in spite that the absolute RMSE values were higher in 50% holdout due to a smaller training set size. The differences were usually below 1% and statistically insignificant according to Wilcoxon test. There were bigger differences between particular datasets, especially between the smallest ones, but over all datasets the average change of RMSE was comparable (r1/r0, r2/r0, $r_{min}/r0$). Thus, it can be said that the MEISR algorithm performed equally well in both testing procedures. There were only bigger differences in the retention at the point c3—on average, the retention was higher by 11% in 50% holdout. This can be explained by the fact that in 50% holdout there are fewer instances in the original training set (50% vs. 90%), and as at the point c2 the instances are sparse and higher percentage of them must remain to allow sufficient instance density to train the model. The differences in $c(r_{min})$ can be bigger because, at that point, the Pareto Front is very flat and very little change in RMSE causes a much bigger change in retention.

The biggest improvement in terms of $RMSE_{tst}$ was observed for 1-NN as the final model (Table A3 and Table A4), where the average $RMSE_{tst}$ decrease was about 3.5% for average retention 62% and 8.6% for average retention 76%.

In case of k-kNN with optimal *k* (Table A5 and Table A6), on average, lower $RMSE_{tst}$ was obtained on the original uncompressed data than at point c1. However, at the point c3 with an average retention rate 85% we were able to reduce $RMSE_{tst}$ on average by 1.5%.

When the final prediction model was an MLP neural network, on average, the RMSEtst decreased by 0.8% at c1 and by 2.5% at rmin (Table A7 and Table A8). At the point c2 (with the strongest compression), the increase of the RMSEtst was about three times lower than for 1-NN and *k*-NN. Thus, the conclusion is the MLP neural network is much less sensitive to instance selection. However, the unquestionable benefit of instance selection, in this case, was a reduction of data sizes and the shortening of the network learning process and thus giving the chance to try many different network configurations in a limited time. Nevertheless, as we have already mentioned, further RMSEtst decrease could be improved by using the MLP network also as the evaluation algorithm on the training set during the evolutionary optimization at the cost of much higher computational complexity.

The real-value instance weighting (Table A9 and Table A10) gave better results (lower Pareto front) only in two areas: for very high compression and for noisy datasets, while, in all the other cases, binary instance selection was better.

The MEISR method outperformed the four ensemble based methods: threshold-based CNN ensemble (TE-C), threshold-based ENN ensemble (TE-E), discretization-based CNN ensemble (DE-C), discretization-based ENN ensemble (DE-E) for the two retention values of 0.5 and 0.25, for which we run the tests (see Table A11 and Table A12).

As we had to chose a single point from the Pareto front for comparison with the DROP3-RT method, in a case that we could not find a point with lower 1 − r${}^{2}$ and stronger compression, we decided to always use a point with stronger compression, even if the 1 − r${}^{2}$ (and RMSE) would be higher (see the results in Table A13 and Table A14).

Comparing with other instance selection methods, the MEISR method allowed for obtaining significantly better results than all the other methods. Only for the largest datasets, the RMSE obtained with DROP3-RT was in several cases lower, but the compression of MEISR was stronger. The statistical tests can be found in Table A15.

### 3.4. Information Distribution, Loss Functions and Error Reduction

As mentioned in the introduction, the entropy used as a measure of information can increase after instance selection in classification tasks and some analogy exists for instance selection for regression problems. Unlike in classification problems, differential entropy used for continuous data (Equation (2)) can also be negative, which means that the information contained in the data is small (which can be thought of as if $2^{H}$ is small, then *H* is negative) [7]. However, unlike only decision boundaries in classification, in regression, each point of the data space matters. For that reason, to assess the instance selection performance in regression, a measure that can show the detail differences between closely located points of the data space will be preferred. Namely, we need to know how well one point can be represented by another closely located point.

Several such measures can be used. The first one is cross entropy, which identifies the difference between two probability distributions *P* and *Q*. It measures the average number of bits needed to identify a point from the set *P*, if a coding scheme from another set *Q* is used. Cross-entropy loss increases when the predicted class frequently differs from the actual label. For discrete data, cross entropy can be written as:

(20) $$CE=-\sum_{i=1}^{n}p\left(y_{i}\right)\log q\left(y_{i}\right).$$

For continuous data, cross entropy can be defined per analogy as

(21) $$CE_{c}=-\int_{Y}p\left(y\right)\log q\left(y\right)dy.$$

However, unlike in classification, in regression problems, we do not usually know the probability distributions. As cross entropy is used as a loss function in machine learning, other loss functions can also be useful to identify the difference between the two distributions. If the difference is high, we can assume that the data contains much noise. In addition, indeed the experimental evaluations confirmed this assumption, as in this case instance selection allowed for significantly decreasing the $RMSE_{tst}$ of the final predictor.

**Figure 5:**
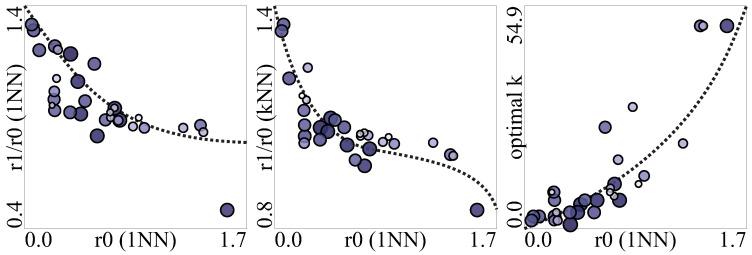
Left and middle: dependency between loss function RMSE(1-NN) and relative $RMSE_{tst}$ (r1/r0) for retention c1. MEISR with 1-NN inner evaluator and 1-NN final regressor for retention c1 (left), MEISR with *k*-NN inner evaluator and *k*-NN final regressor center) and *k* = optimal *k*. Larger and darker circles stand for datasets with higher number of instances. Right: dependency between optimal *k* and $RMSE_{tst}$. Darker and bigger points represent larger datasets.

In regression tasks, the most common loss function is root mean square error (RMSE). Since this measure was used in the optimization, we will also use it to show the dependencies. The loss functions r0 for 1-NN and the relative $RMSE_{tst}$ (r1/r0) obtained for retention = c1 are shown in Figure 5, where r0 is the $RMSE_{tst}$ without instance selection and r1 is the $RMSE_{tst}$ obtained for compression c1. The Pearson correlation coefficients between these variables were −0.782 for 1-NN and −0.728 for *k*-NN with optimal *k*.

We also observed that the optimal *k* on the selected subset was frequently different than before instance selection, and it tended to converge to the range of 5 to 7. That is, if the optimal *k* was 2 before instance selection it may increase after and if it was 11 before it is more likely to decrease. It can be explained, as after the selection fewer instances remained in the dataset, so the distances between them were bigger and frequently the previous closest neighbor of the examined instance no longer existed and thus it had to be replaced by the average of some further still existing instances. On the other hand, a high value of the optimal *k* is characteristic for noisy datasets, as an average value of several neighbors is needed to mask the noise. The correlation is shown in Figure 5. Instance selection removes the outlier and noisy instances, thus no longer so many neighbors are required to mask the detrimental effect of noise. Therefore, in the cases, where the optimal *k* was above 11, we used *k* = 11 because otherwise the optimal *k* would decrease anyway during the instance selection process and starting the optimization with *k* = 11 allowed for obtaining lower $RMSE_{trn}$ and $RMSE_{tst}$ on the selected subset. The dependency between r0 for 1-NN and the *k* value that was optimal on the original datasets (orgK in Table A1) characterized by correlation coefficient 0.844 is also shown in Figure 5.

### 3.5. Computational Complexity

In this case, contrary to popular belief, the evolutionary algorithm based solution does not have to be more computationally expensive than the non-evolutionary ones.

The MEISR instance selection process can be decomposed into two steps:

1. The first step—calculating the distance matrices (see Section 2.3) has the complexity $O(n^{2})$.
2. The second step—running the evolutionary optimization has the complexity O(nlogn)—because of the increasing number of epochs with dataset size.

One operation in the second step takes longer than in the first step. For small datasets, the second step is dominant, but, for the big ones, the first step. The measurements showed that for 900 instances the first step took about 10% of the total time, but, for 36,690 instances, it took about 65%.

Most of the non-evolutionary instance selection algorithms must also calculate the distance matrix or other equivalent matrix. Their complexity is between $O(n^{2})$ (ENN, RHMC, ELH) and $O(n^{3})$ (DROP1-5, GE, RNGE) [22]. We really observed that, for big datasets, the instance selection time with DROP3 grows much faster than with MEISR.

In the first step, the distances between each instances in the training set are first calculated in $O(n^{2})$ and then they are sorted in O(nlogn), so the complexity is $O(n^{2})$—the higher of the two. The time spent on calculating the distance matrix also grows with the number of attributes.

The second step consists of several operations. Calculating the fitness function has the complexity O(n) because the output value of *n* instances must be obtained, where n=N is the number of instances in the original training set. Obtaining the output value requires reading on average *k* non-zero positions from sorted output value arrays, where *k* is the number of nearest neighbors in the *k*-NN algorithm, which assuming a reduction rate of 50% requires reading 2k entries and calculating the average of them. The time spent in this step grows with the number of *k*, but much slower than linearly because also other operations are performed in this step, which do not depend on *k*, or depend but weaker than linearly, as crossover, mutation, and selection. The proportions of time spent at each step depend on particular software implementation. The experimental measurements confirmed that the complexity of the step can be considered O(nlogn).

In a practical software implementation, there is also a third-factor consuming time: the constant operations independent on the data, as calling functions, creating objects, etc. This factor is most significant with very small datasets and this is the reason that the times per one instance (two last columns in Table A16) are higher for the smallest dataset than for some of the following datasets.

**Figure 6:**
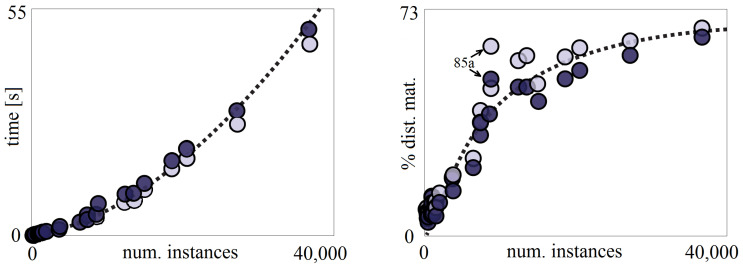
Left: MEISR running time as a function of number of instances in the original training dataset. Right: Percentage of MEISR running time used to calculate the distance matrix used by *k*-NN. Light circles denote 1-NN as the inner evaluator and dark circles *k*-NN with optimal *k*.

Figure 6 shows the dependency of the MEISR running time on the number of instances (left) for 1-NN and *k*-NN with optimal *k* and the percentage of the running time used to calculate the distance matrix used by *k*-NN. The two points marked as “85a” denote the tick dataset, which has 85 attributes (more than other datasets) and, for that reason, there is so high cost of calculating the distance matrix for this case. The measurements were performed using our software (available from the online supplementary resources) on a computer with two Xeon E5-2696v2 processors. Detailed values can be found in Table A16).

### 3.6. Performance Metrics for Multi-Objective Evolutionary Algorithms

To measure the behavior of the NSGA-II algorithm used within the instance selection process, we provide the performance metrics for multi-objective evolutionary algorithms for binary instance selection with *k*-NN with optimal *k* (for other sets of experiments, the metrics were very similar).

However, first, it must be emphasized that the metrics express only the performance of the evolutionary algorithm in terms of data reduction and $RMSE_{trn}$ on the training set and not of the whole process of instance selection, where the final objectives are data reduction and $RMSE_{tst}$ on the test set. Second, as it was discussed that we had to stop the optimization early to prevent over-fitting and, for that reason, we could not achieve performance metrics that were as good as could be obtained if the target objectives would be optimized directly.

We calculated the following popular metrics: Ratio of Non-dominated Individuals—RNI [61], Inverted Generational Distance—IGD [62] (which expresses closeness of the solutions to the true Pareto front), Uniform Distribution—UD [61] (which expresses distribution of the solutions, with the σ for UD metric set to 0.025), Maximum Spread—MS [63] (spread of the solutions) and HyperVolume indicator—HV [64,65] (which applies to several of listed categories, with a reference point for HV metric set to +5%).

The obtained values of metrics can be summed up as follows:

- The RNI values are high, average 0.560, which means that more than 50% of the population formed a Pareto front.
- The IGD values are low, average: 0.014 (lower = better), which means that the results were always close to the optimal Pareto front.
- The UD values are low, average: 0.234 (lower = better), which means that most of the solutions were properly spread.
- The MS values are high, average: 0.833 (higher = better), which means that obtained Pareto fronts are wide in range in comparison to optimal Pareto front.
- The average HV values are 0.039, which means that the values of $RMSE_{trn}$, even for high compression, did not increase significantly, which is good because lower RMSE is preferred and the very low compression is not present in the results, which is also good because the desired RMSE has already been achieved with stronger compression and thus HV covers a satisfactory part of the objectives’ area.

## 4. Conclusions

We presented and experimentally evaluated an instance selection method applied for regression tasks, which uses the *k*-NN algorithm for error evaluation a multi-objective evolutionary algorithm (NSGA-II in the current implementation) for directing the search process. Different aspects of the solution were discussed, many improvements were proposed and different tests were performed. The following main conclusions can be drawn from this work:

- A key advantage of the MEISR method is that we obtain several solutions from the Pareto front, and can choose one of them depending on our preferences of the RMSE–compression balance. As the solutions create a Pareto front, each of them is best for a given balance between RMSE and compression, as explained in Section 2.2. If someone is not sure which solution to choose, then we suggest the solution with the lowest RMSE on the training set ($RMSE_{trn}$) for the purpose for machine learning and the solution (c2,r2) for analyzing the properties of the data, as explained in Figure 2 and in the text following it. This is valid for every dataset, as this is the characteristic feature of the multi-objective optimization itself and it is not dependent on the dataset properties.
- *k*-NN is very well suited as the inner evaluation algorithm because of its speed—the distance matrix has to be calculated and sorted only once and then the prediction is extremely fast. This makes the computational cost of the method comparable to the cost on non-evolutionary instance selection algorithms (and some of them, as the DROP family have even higher cost), while the results are usually better (see Section 3.5, Figure 6 and Table A16 for details).
- We were frequently able to preserve all the useful information in the dataset for the purpose of predictive model performance while reducing it size by about one third (see columns 4 and 5 in Table A3, Table A4, Table A5, Table A6, Table A7 and Table A8 and Figure 4).
- Proper initialization of the population accelerates the instance selection process and helps to find the desired solutions (see Section 2.4).
- The best results in terms of RMSE-compression balance can be obtained if the inner evaluation algorithm is the same as the final predictor [24]. For that reason, we used 1-NN as the internal regressor when 1-NN was used as the final prediction model and *k*-NN with optimal *k* as the internal regressor when *k*-NN with optimal *k* was used as the final prediction model.
- Although our previous experiments showed [24] that when an MLP neural network as the final predictor better results were achieved if also the internal regressor was an MLP network, it would be very time-consuming, especially for bigger datasets, as the MLP network has to be trained each time (at least on the part of the data close to the currently evaluated point). Thus, we decided to use *k*-NN with optimal *k* as the internal regressor for the MLP neural network.
- We noticed that there are two areas where real-value instance weighting can provide better results than binary instance selection: for very high compression, where it was usually able to achieve lower RMSE and for noisy datasets, which required high *k* value in *k*-NN (see Table A10).
- The obtained $RMSE_{tst}$ with the *k*-NN algorithm for a given point on the Pareto front (for a given compression) can be approximately assessed by the measures of how well one instances can be substituted by other, e.g., the loss functions of cross entropy or RMSE. The lower values of the loss function correspond with lower possible decrease $RMSE_{tst}$.
- The MEISR method achieved better results than the other 12 instance selection methods for regression, for which we were able to obtain the experimental results to perform the comparison (see Table A12 and Table A14)
- We can observe that the “front” is less steep for the test set than for the training set. This is the compression strength grows and the RMSE increases on average slower on the test set than on the corresponding training set solutions (when moving from right to left in Figure 2, the green points are getting closer to the orange ones). This allows for choosing a point with higher compression, as the $RMSE_{tst}$ on that point is likely to grow less than $RMSE_{trn}$
- There were not significant differences between the algorithm performance tested with 50% holdout and 10-fold cross-validation. Only retention at the point c2 was on average higher 11% in the first case, as there were already fewer instances in the original training set.

We have also noticed two areas of possible improvement and we are going to investigate them in our future work:

- A single front covered all solutions of interest in many cases, but not in all. One of the reasons is the tendency of multi-objective evolutionary algorithms to not cover the areas on the ends of the front, as was discussed and some solutions were proposed i.e., in [66,67]. The second reason is that the front extends gradually during the optimization and, to prevent over-fitting, we must stop the instance selection process before the front is fully extended.
- Experimental comparison with other instance selection methods showed that, for the small and medium size datasets, the MEISR method has the greatest advantage over other methods (see Table A12 and Table A14). However, for the largest datasets, while still the compression was always stronger, the obtained $1-r^{2}$ began to became similar to that of DROP3-RT method. Although MEISR optimized RMSE and $1-r^{2}$ was used only for comparison and the relation between RMSE and $1-r^{2}$ is not linear, using $1-r^{2}$ as the objective on the training set would most likely improve the results. This would be true also for the smaller datasets and the tendency would remain. A well known issue here is that, for genetic algorithms with longer chromosomes, the convergence is more difficult. Thus, we are going to investigate the possibilities of alternative encoding of the instances to limit the chromosome length.

To summarize: the presented method of instance selection in regression tasks has proved to work effectively and has several advantages. Thus, we believe it can be helpful for researchers and practitioners in industry. Moreover, there is probably still room for further improvements. 

## Appendix A. Detailed Experimental Results

This section contains tables with dataset properties and detailed experimental results. The following symbols are used:

- r0, r1, r2, r${}_{min}$—absolute $RMSE_{tst}$ (see Figure 2 for details),
- r1/r0, r2/r0, r${}_{min}$/r0—relative RMSE (see Figure 2 for details),
- c1, c2—retention (see Figure 2 for details),
- c(r)—retention for specified r,
- $r_{min}=min\left(r0,r1,r2,r3\right)$—the lowest obtained $RMSE_{tst}$,
- IEA—Inner Evaluation Algorithm,
- FPA—Final Prediction Algorithm,
- BIS—Binary Instance Selection,
- RIS—Real-value Instance Selection,
- Dr2—relative 1 −$r^{2}$ with DROP3-RT,
- Mr2—relative 1 −$r^{2}$ with MEISR,
- Dc1—retention rate with DROP3-RT,
- Mc1—retention rate with MEISR,
- TE-C, TE-E, DE-C, DE-E, MEISR (in Table A11 and Table A12)—$RMSE_{tst}$ for these algorithms,
- 10-CV—10-fold cross-validation,
- 50%H—50% holdout; (50% training set, and 50% in test set)—average of 10 runs.

Results for each experimental case, averaged for all datasets, are presented the Table A2 and the statistical significance test results in Table A15. Results for particular configurations fo the MEISR algorithm are presented in Table A3, Table A4, Table A5, Table A6, Table A7, Table A8, Table A9 and Table A10. Result comparison with other algorithms is presented in Table A11, Table A12, Table A13 and Table A14.

The calculation times is and its particular components are presented in Table A16.

**Table A1:** Datasets used in the experiments and their properties: number of instances (Ints.), number of attributes (Attr.), the optimal *k*—the *k* in *k*-NN that gives the lowest $RMSE_{tst}$ (orgK) and the *k* used in the experiments as optimal *k* (optK) for the reason explained further in the text.

Dataset	Inst.	Attr.	orgK	optK
mach. CPU	209	6	1	1
baseball	337	16	7	7
dee	365	6	7	7
autoMPG8	392	7	6	6
autoMPG6	392	5	4	4
ele-1	495	2	11	11
forestFires	517	12	50	11
stock	950	9	3	3
steel	960	12	4	4
laser	993	4	3	3
concrete	1030	8	4	4
treasury	1049	15	3	3
mortgage	1049	15	2	2
friedman	1200	5	7	7
wizmir	1461	9	7	7
wankara	1609	9	9	9
plastic	1650	2	3	11
quake	2178	3	50	11
anacalt	4052	7	2	2
abalone	4177	8	13	11
delta-ail	7128	5	17	11
puma32h	8191	32	21	11
compactiv	8192	21	2	2
delta-elv	9516	6	35	11
tic	9822	85	50	11
ailerons	13,750	40	10	10
pole	14,998	26	4	4
elevators	16,598	18	8	8
california	20,640	8	9	9
house	22,784	16	11	11
mv	40,767	10	9	9

**Table A2:** Averaged results for experiments presented in Table A3, Table A4, Table A5, Table A6, Table A7, Table A8, Table A9 and Table A10.

IEA	FPA	Validation	r0	r1	r1/r0	c1	r2	r2/r0	c2	Details
1-NN (BIS)	1-NN (BIS)	50%H	0.604	0.537	0.955	0.619	0.646	1.176	0.246	see Table A3
1-NN (BIS)	1-NN (BIS)	10-CV	0.573	0.503	0.960	0.627	0.594	1.175	0.214	see Table A4
*k*-NN (BIS)	*k*-NN (BIS)	50%H	0.476	0.486	1.050	0.505	0.516	1.163	0.249	see Table A5
*k*-NN (BIS)	*k*-NN (BIS)	10-CV	0.446	0.453	1.056	0.497	0.487	1.203	0.227	see Table A6
*k*-NN (BIS)	MLP	50%H	0.427	0.424	0.994	0.505	0.458	1.076	0.249	see Table A7
*k*-NN (BIS)	MLP	10-CV	0.393	0.391	0.992	0.497	0.418	1.068	0.227	see Table A8
*k*-NN (RIS)	*k*-NN (RIS)	50%H	0.476	0.496	1.116	0.557	0.555	1.307	0.185	see Table A9
*k*-NN (RIS)	*k*-NN (RIS)	10-CV	0.448	0.465	1.113	0.532	0.508	1.282	0.165	see Table A10

**Table A3:** Experimental results for MEISR with 50%H, IEA: 1-NN (BIS), FPA: 1-NN (BIS).

Dataset	r0	r1	r1/r0	c1	r2	r2/r0	c2	r${}_{\min}$	r${}_{\min}$/r0	c(r${}_{\min}$)
mach. CPU	0.450	0.517	1.149	0.459	0.541	1.202	0.249	0.428	0.951	0.910
baseball	0.758	0.746	0.984	0.609	0.789	1.041	0.668	0.746	0.984	0.609
dee	0.561	0.502	0.894	0.433	0.561	0.999	0.532	0.502	0.894	0.433
autoMPG8	0.478	0.450	0.942	0.519	0.509	1.065	0.199	0.450	0.942	0.519
autoMPG6	0.464	0.464	1.001	0.653	0.691	1.491	0.203	0.451	0.973	0.925
ele-1	0.690	0.670	0.970	0.628	0.708	1.025	0.596	0.670	0.970	0.628
forestFires	1.669	0.899	0.539	0.413	0.902	0.541	0.201	0.899	0.539	0.413
stock	0.136	0.158	1.162	0.450	0.217	1.596	0.168	0.136	1.000	1.000
steel	0.359	0.404	1.124	0.480	0.416	1.158	0.391	0.359	1.000	1.000
laser	0.293	0.312	1.064	0.516	0.343	1.170	0.208	0.284	0.969	0.898
concrete	0.639	0.677	1.060	0.664	0.825	1.292	0.201	0.639	1.000	1.000
treasury	0.092	0.095	1.033	0.530	0.145	1.576	0.180	0.090	0.978	0.902
mortgage	0.071	0.082	1.151	0.453	0.112	1.572	0.309	0.071	1.000	1.000
friedman	0.510	0.467	0.915	0.735	0.647	1.268	0.156	0.467	0.915	0.735
wizmir	0.244	0.245	1.003	0.726	0.316	1.293	0.192	0.244	1.000	1.000
wankara	0.239	0.229	0.959	0.727	0.295	1.236	0.209	0.229	0.959	0.727
plastic	0.634	0.518	0.817	0.719	0.608	0.959	0.286	0.518	0.817	0.719
quake	1.349	1.124	0.833	0.723	1.348	0.999	0.143	1.124	0.833	0.723
anacalt	0.263	0.272	1.036	0.521	0.304	1.157	0.217	0.248	0.944	0.886
abalone	0.900	0.771	0.857	0.718	0.926	1.029	0.185	0.771	0.857	0.718
delta-ail	0.733	0.691	0.943	0.706	0.784	1.069	0.208	0.691	0.943	0.706
puma32h	1.227	1.036	0.844	0.716	1.279	1.042	0.213	1.036	0.844	0.716
compactiv	0.303	0.344	1.135	0.396	0.384	1.267	0.312	0.303	1.000	1.000
delta-elv	0.826	0.709	0.859	0.719	0.901	1.091	0.165	0.709	0.859	0.719
tic	1.348	1.111	0.824	0.719	1.349	1.001	0.189	1.111	0.824	0.719
ailerons	0.685	0.601	0.878	0.720	0.748	1.092	0.144	0.601	0.878	0.720
pole	0.271	0.288	1.061	0.716	0.371	1.367	0.205	0.271	1.000	0.925
elevators	0.719	0.659	0.916	0.719	0.940	1.307	0.171	0.659	0.916	0.719
california	0.681	0.612	0.899	0.716	0.766	1.125	0.163	0.612	0.899	0.716
house	0.893	0.787	0.881	0.717	0.983	1.101	0.199	0.787	0.881	0.717
mv	0.235	0.208	0.884	0.677	0.312	1.325	0.162	0.208	0.884	0.677
average	0.604	0.537	0.955	0.619	0.646	1.176	0.246	0.526	0.918	0.777

**Table A4:** Experimental results for MEISR with 10-CV, IEA: 1-NN (BIS), FPA: 1-NN (BIS).

Dataset	r0	r1	r1/r0	c1	r2	r2/r0	c2	r${}_{\min}$	r${}_{\min}$/r0	c(r${}_{\min}$)
mach. CPU	0.351	0.407	1.159	0.385	0.426	1.212	0.216	0.351	1.000	0.914
baseball	0.727	0.639	0.878	0.662	0.654	0.899	0.559	0.639	0.878	0.662
dee	0.555	0.450	0.811	0.652	0.510	0.918	0.401	0.450	0.811	0.652
autoMPG8	0.429	0.387	0.904	0.685	0.483	1.127	0.155	0.387	0.904	0.685
autoMPG6	0.407	0.424	1.042	0.698	0.506	1.242	0.150	0.401	0.985	0.920
ele-1	0.689	0.640	0.929	0.678	0.666	0.966	0.619	0.640	0.929	0.678
forestFires	1.548	0.768	0.496	0.344	0.796	0.514	0.191	0.768	0.496	0.344
stock	0.112	0.132	1.175	0.629	0.190	1.692	0.155	0.112	1.000	0.918
steel	0.348	0.317	0.911	0.469	0.402	1.154	0.369	0.317	0.911	0.469
laser	0.231	0.276	1.192	0.467	0.306	1.324	0.221	0.231	1.000	0.911
concrete	0.533	0.595	1.117	0.696	0.768	1.441	0.155	0.533	1.000	1.000
treasury	0.069	0.087	1.259	0.662	0.118	1.693	0.159	0.069	1.000	0.916
mortgage	0.054	0.069	1.284	0.545	0.096	1.784	0.287	0.054	1.000	0.926
friedman	0.462	0.443	0.959	0.692	0.595	1.288	0.148	0.443	0.959	0.692
wizmir	0.230	0.211	0.919	0.693	0.301	1.311	0.160	0.211	0.919	0.693
wankara	0.225	0.218	0.966	0.692	0.289	1.285	0.160	0.218	0.966	0.692
plastic	0.617	0.543	0.879	0.682	0.607	0.983	0.212	0.543	0.879	0.682
quake	1.344	1.151	0.856	0.685	1.349	1.003	0.157	1.151	0.856	0.685
anacalt	0.227	0.227	1.000	0.449	0.286	1.260	0.186	0.222	0.977	0.887
abalone	0.915	0.773	0.845	0.685	0.880	0.962	0.155	0.773	0.845	0.685
delta-ail	0.716	0.628	0.877	0.685	0.738	1.031	0.159	0.628	0.877	0.685
puma32h	1.212	1.024	0.845	0.689	1.212	1.000	0.161	1.024	0.845	0.689
compactiv	0.254	0.299	1.177	0.429	0.297	1.172	0.237	0.235	0.926	0.847
delta-elv	0.828	0.705	0.851	0.685	0.837	1.010	0.157	0.705	0.851	0.685
tic	1.366	1.130	0.827	0.685	1.330	0.973	0.156	1.130	0.827	0.685
ailerons	0.657	0.584	0.889	0.688	0.709	1.079	0.160	0.584	0.889	0.688
pole	0.244	0.258	1.055	0.686	0.353	1.443	0.157	0.244	1.000	0.922
elevators	0.686	0.638	0.930	0.687	0.764	1.114	0.162	0.638	0.930	0.687
california	0.654	0.596	0.911	0.686	0.718	1.099	0.158	0.596	0.911	0.686
house	0.872	0.775	0.888	0.684	0.926	1.061	0.159	0.775	0.888	0.684
mv	0.210	0.197	0.941	0.686	0.291	1.387	0.158	0.197	0.941	0.686
average	0.573	0.503	0.960	0.627	0.594	1.175	0.214	0.493	0.910	0.740

**Table A5:** Experimental results for MEISR with 50%H, IEA: *k*-NN (BIS), FPA: *k*-NN (BIS).

Dataset	r0	r1	r1/r0	c1	r2	r2/r0	c2	r${}_{\min}$	r${}_{\min}$/r0	c(r${}_{\min}$)
mach. CPU	0.450	0.517	1.149	0.437	0.541	1.202	0.249	0.441	0.980	0.877
baseball	0.648	0.671	1.035	0.409	0.718	1.108	0.287	0.648	1.000	0.409
dee	0.472	0.454	0.962	0.378	0.462	0.979	0.314	0.442	0.937	0.912
autoMPG8	0.422	0.412	0.976	0.428	0.481	1.140	0.299	0.407	0.964	0.936
autoMPG6	0.411	0.398	0.968	0.489	0.452	1.100	0.261	0.377	0.917	0.939
ele-1	0.584	0.562	0.962	0.409	0.589	1.009	0.321	0.562	0.962	0.409
forestFires	0.901	0.847	0.940	0.324	0.870	0.966	0.230	0.847	0.940	0.324
stock	0.129	0.148	1.147	0.542	0.168	1.302	0.293	0.124	0.961	0.962
steel	0.364	0.391	1.076	0.481	0.490	1.348	0.247	0.364	1.000	0.912
laser	0.274	0.284	1.038	0.472	0.324	1.184	0.239	0.258	0.943	0.941
concrete	0.576	0.631	1.096	0.497	0.687	1.193	0.205	0.576	1.000	0.922
treasury	0.079	0.086	1.093	0.502	0.126	1.594	0.255	0.079	1.000	1.000
mortgage	0.065	0.082	1.258	0.517	0.137	2.101	0.172	0.065	1.000	0.941
friedman	0.375	0.410	1.092	0.506	0.490	1.305	0.200	0.375	1.000	0.899
wizmir	0.199	0.215	1.081	0.384	0.225	1.131	0.205	0.199	1.000	1.000
wankara	0.185	0.199	1.076	0.503	0.224	1.211	0.210	0.185	1.000	1.000
plastic	0.463	0.460	0.994	0.491	0.458	0.989	0.211	0.460	0.994	0.491
quake	1.024	1.008	0.984	0.621	1.030	1.005	0.215	1.008	0.984	0.621
anacalt	0.242	0.254	1.052	0.484	0.286	1.184	0.260	0.242	1.000	1.000
abalone	0.713	0.704	0.987	0.655	0.721	1.011	0.197	0.704	0.987	0.887
delta-ail	0.582	0.588	1.010	0.676	0.688	1.181	0.187	0.582	1.000	0.905
puma32h	0.910	0.918	1.008	0.583	0.932	1.024	0.220	0.910	1.000	1.000
compactiv	0.281	0.316	1.125	0.426	0.337	1.200	0.243	0.281	1.000	1.000
delta-elv	0.626	0.624	0.996	0.687	0.628	1.003	0.266	0.624	0.996	1.000
tic	1.011	0.997	0.986	0.683	1.007	0.996	0.224	0.997	0.986	0.683
ailerons	0.522	0.533	1.021	0.523	0.568	1.088	0.272	0.522	1.000	0.932
pole	0.244	0.270	1.107	0.516	0.301	1.234	0.302	0.244	1.000	0.933
elevators	0.586	0.604	1.030	0.534	0.643	1.097	0.287	0.586	1.000	1.000
california	0.548	0.566	1.033	0.535	0.446	0.814	0.299	0.548	1.000	0.948
house	0.712	0.724	1.017	0.566	0.764	1.073	0.287	0.712	1.000	0.901
mv	0.160	0.198	1.236	0.384	0.205	1.280	0.267	0.160	1.000	1.000
average	0.476	0.486	1.050	0.505	0.516	1.163	0.249	0.468	0.986	0.861

**Table A6:** Experimental results for MEISR with 10-CV, IEA: *k*-NN (BIS), FPA: *k*-NN (BIS).

Dataset	r0	r1	r1/r0	c1	r2	r2/r0	c2	r${}_{\min}$	r${}_{\min}$/r0	c(r${}_{\min}$)
mach. CPU	0.351	0.370	1.053	0.436	0.410	1.168	0.213	0.339	0.964	0.852
baseball	0.584	0.582	0.998	0.450	0.649	1.112	0.217	0.582	0.998	0.450
dee	0.424	0.427	1.008	0.403	0.439	1.035	0.229	0.418	0.986	0.928
autoMPG8	0.372	0.401	1.078	0.458	0.422	1.134	0.218	0.364	0.978	0.927
autoMPG6	0.366	0.382	1.043	0.424	0.393	1.074	0.228	0.351	0.921	0.944
ele-1	0.584	0.557	0.954	0.533	0.585	1.002	0.367	0.557	0.954	0.533
forestFires	0.864	0.725	0.839	0.340	0.726	0.840	0.162	0.725	0.839	0.340
stock	0.105	0.124	1.181	0.486	0.145	1.386	0.340	0.104	0.990	0.954
steel	0.323	0.330	1.022	0.444	0.421	1.302	0.224	0.323	1.000	0.934
laser	0.204	0.210	1.031	0.435	0.261	1.280	0.208	0.195	0.955	0.926
concrete	0.521	0.549	1.054	0.488	0.648	1.244	0.193	0.521	1.000	0.929
treasury	0.058	0.077	1.321	0.486	0.103	1.768	0.234	0.058	1.000	0.927
mortgage	0.045	0.059	1.304	0.489	0.107	2.387	0.156	0.045	1.000	0.923
friedman	0.340	0.365	1.073	0.472	0.441	1.297	0.162	0.340	1.000	0.947
wizmir	0.178	0.188	1.060	0.470	0.231	1.297	0.214	0.178	1.000	0.779
wankara	0.167	0.183	1.098	0.497	0.220	1.315	0.160	0.167	1.000	1.000
plastic	0.468	0.453	0.969	0.446	0.466	0.996	0.212	0.453	0.969	0.446
quake	1.025	1.008	0.983	0.685	1.025	1.001	0.219	1.008	0.983	0.685
anacalt	0.212	0.215	1.013	0.440	0.282	1.330	0.174	0.209	0.988	0.684
abalone	0.702	0.709	1.011	0.682	0.750	1.068	0.158	0.688	0.981	0.801
delta-ail	0.560	0.579	1.033	0.686	0.604	1.078	0.158	0.560	1.000	0.835
puma32h	0.896	0.908	1.014	0.461	0.920	1.027	0.222	0.896	1.000	1.000
compactiv	0.231	0.280	1.209	0.461	0.286	1.238	0.275	0.231	1.000	1.000
delta-elv	0.610	0.620	1.016	0.684	0.628	1.029	0.225	0.610	1.000	1.000
tic	1.015	0.995	0.980	0.684	1.011	0.996	0.186	0.995	0.980	0.684
ailerons	0.504	0.519	1.031	0.473	0.550	1.092	0.263	0.504	1.000	0.934
pole	0.214	0.241	1.125	0.491	0.261	1.219	0.307	0.214	1.000	0.947
elevators	0.559	0.581	1.040	0.448	0.622	1.112	0.226	0.559	1.000	1.000
california	0.527	0.546	1.037	0.488	0.571	1.083	0.332	0.527	1.000	0.944
house	0.687	0.707	1.029	0.483	0.737	1.074	0.276	0.687	1.000	0.894
mv	0.140	0.159	1.136	0.492	0.183	1.311	0.273	0.140	1.000	1.000
average	0.446	0.453	1.056	0.497	0.487	1.203	0.227	0.437	0.984	0.843

**Table A7:** Experimental results for MEISR with 50%H, IEA: *k*-NN (BIS), FPA: MLP.

Dataset	r0	r1	r1/r0	c1	r2	r2/r0	c2	r${}_{\min}$	r${}_{\min}$/r0	c(r${}_{\min}$)
mach. CPU	0.457	0.491	1.076	0.437	0.570	1.247	0.249	0.457	1.000	0.877
baseball	0.618	0.659	1.067	0.409	0.763	1.235	0.287	0.618	1.000	0.409
dee	0.439	0.453	1.033	0.378	0.500	1.141	0.314	0.428	0.976	0.912
autoMPG8	0.366	0.343	0.937	0.428	0.432	1.182	0.299	0.366	1.000	1.000
autoMPG6	0.367	0.344	0.938	0.489	0.416	1.132	0.261	0.344	0.938	0.939
ele-1	0.554	0.512	0.923	0.409	0.585	1.056	0.321	0.512	0.923	0.409
forestFire	1.084	1.026	0.946	0.324	1.124	1.037	0.230	1.026	0.946	0.324
stock	0.195	0.190	0.974	0.542	0.198	1.011	0.293	0.190	0.974	0.962
steel	0.251	0.219	0.872	0.481	0.224	0.893	0.247	0.219	0.872	0.481
laser	0.204	0.203	0.994	0.472	0.203	0.995	0.239	0.203	0.994	1.000
concrete	0.407	0.423	1.039	0.497	0.449	1.103	0.205	0.407	1.000	1.000
treasury	0.077	0.076	0.985	0.502	0.080	1.050	0.255	0.076	0.985	1.000
mortgage	0.070	0.069	0.981	0.517	0.079	1.127	0.172	0.066	0.941	0.941
friedman	0.301	0.300	0.997	0.506	0.347	1.152	0.200	0.298	0.989	0.899
wizmir	0.095	0.096	1.007	0.384	0.099	1.042	0.205	0.095	1.000	1.000
wankara	0.100	0.098	0.980	0.503	0.102	1.023	0.210	0.096	0.961	0.843
plastic	0.435	0.437	1.004	0.491	0.442	1.016	0.211	0.435	1.000	0.491
quake	1.000	0.999	0.999	0.621	1.054	1.054	0.215	0.999	0.999	0.621
anacalt	0.223	0.199	0.891	0.484	0.251	1.124	0.260	0.199	0.891	0.484
abalone	0.652	0.642	0.984	0.655	0.646	0.991	0.197	0.642	0.984	0.887
delta-ail	0.554	0.552	0.997	0.676	0.551	0.996	0.187	0.550	0.993	0.905
puma32h	0.368	0.406	1.104	0.583	0.473	1.286	0.220	0.368	1.000	1.000
compactiv	0.155	0.158	1.019	0.426	0.164	1.055	0.243	0.155	1.000	1.000
delta-elv	0.600	0.590	0.982	0.687	0.614	1.022	0.266	0.588	0.980	0.910
tic	1.019	1.014	0.995	0.683	1.052	1.033	0.224	1.014	0.995	0.683
ailerons	0.414	0.425	1.026	0.523	0.447	1.080	0.272	0.414	1.000	1.000
pole	0.241	0.251	1.041	0.516	0.282	1.170	0.302	0.241	1.000	1.000
elevators	0.682	0.680	0.997	0.534	0.706	1.036	0.287	0.677	0.993	0.922
california	0.532	0.531	0.998	0.535	0.550	1.034	0.299	0.532	1.000	1.000
house	0.712	0.719	1.011	0.566	0.728	1.023	0.287	0.712	1.000	1.000
mv	0.055	0.055	1.002	0.384	0.055	1.008	0.267	0.055	1.000	1.000
average	0.427	0.424	0.994	0.505	0.458	1.076	0.249	0.419	0.979	0.835

**Table A8:** Experimental results for MEISR with 10-CV, IEA: *k*-NN (BIS), FPA: MLP.

Dataset	r0	r1	r1/r0	c1	r2	r2/r0	c2	r${}_{\min}$	r${}_{\min}$/r0	c(r${}_{\min}$)
mach. CPU	0.348	0.379	1.089	0.436	0.438	1.261	0.213	0.378	1.000	1.000
baseball	0.626	0.656	1.048	0.450	0.750	1.198	0.217	0.643	1.000	1.000
dee	0.407	0.412	1.012	0.403	0.470	1.153	0.229	0.404	0.992	0.928
autoMPG8	0.354	0.336	0.948	0.458	0.421	1.187	0.218	0.334	0.942	0.927
autoMPG6	0.357	0.330	0.924	0.424	0.407	1.141	0.228	0.326	0.914	0.944
ele-1	0.531	0.488	0.920	0.533	0.552	1.040	0.367	0.486	0.917	0.533
forestFires	0.735	0.699	0.952	0.340	0.745	1.014	0.162	0.691	0.940	0.340
stock	0.178	0.171	0.958	0.486	0.181	1.016	0.340	0.168	0.945	0.954
steel	0.225	0.198	0.882	0.444	0.199	0.885	0.224	0.196	0.872	0.934
laser	0.164	0.162	0.988	0.435	0.165	1.011	0.208	0.160	0.975	0.926
concrete	0.379	0.386	1.017	0.488	0.416	1.097	0.193	0.379	1.000	0.929
treasury	0.074	0.072	0.970	0.486	0.077	1.042	0.234	0.072	0.968	0.927
mortgage	0.055	0.054	0.975	0.489	0.060	1.088	0.156	0.053	0.958	0.923
friedman	0.318	0.315	0.992	0.472	0.368	1.159	0.162	0.313	0.985	0.947
wizmir	0.085	0.086	1.013	0.470	0.087	1.027	0.214	0.085	1.000	1.000
wankara	0.097	0.095	0.978	0.497	0.097	1.004	0.160	0.094	0.968	1.000
plastic	0.437	0.433	0.990	0.446	0.434	0.995	0.212	0.433	0.990	0.446
quake	1.000	0.992	0.992	0.685	1.017	1.017	0.219	0.992	0.992	0.685
anacalt	0.201	0.181	0.898	0.440	0.220	1.096	0.174	0.180	0.894	0.684
abalone	0.651	0.650	0.998	0.682	0.655	1.006	0.158	0.649	0.997	0.801
delta-ail	0.552	0.551	0.997	0.686	0.555	1.005	0.158	0.548	0.993	0.835
puma32h	0.338	0.373	1.103	0.461	0.433	1.283	0.222	0.371	1.000	1.000
compactiv	0.152	0.155	1.018	0.461	0.158	1.042	0.275	0.153	1.000	1.000
delta-elv	0.599	0.598	0.998	0.684	0.604	1.009	0.225	0.596	0.995	1.000
tic	1.017	1.017	1.000	0.684	1.068	1.050	0.186	1.011	0.993	0.684
ailerons	0.402	0.409	1.018	0.473	0.418	1.041	0.263	0.407	1.000	1.000
pole	0.255	0.265	1.038	0.491	0.303	1.188	0.307	0.262	1.000	1.000
elevators	0.344	0.347	1.011	0.448	0.348	1.013	0.226	0.347	1.000	1.000
california	0.532	0.531	0.997	0.488	0.532	0.999	0.332	0.524	0.985	0.944
house	0.710	0.714	1.005	0.483	0.722	1.017	0.276	0.710	1.000	1.000
mv	0.055	0.055	1.005	0.492	0.056	1.009	0.273	0.055	0.991	1.000
average	0.393	0.391	0.992	0.497	0.418	1.068	0.227	0.388	0.974	0.880

**Table A9:** Experimental results for MEISR with 50%H, IEA: *k*-NN (RIS), FPA: *k*-NN (RIS).

Dataset	r0	r1	r1/r0	c1	r2	r2/r0	c2
mach. CPU	0.450	0.462	1.027	0.402	0.485	1.079	0.230
baseball	0.648	0.661	1.020	0.650	0.820	1.265	0.114
dee	0.472	0.471	0.998	0.429	0.577	1.222	0.148
autoMPG8	0.422	0.460	1.090	0.525	0.546	1.295	0.166
autoMPG6	0.411	0.442	1.076	0.402	0.511	1.243	0.176
ele-1	0.584	0.569	0.974	0.533	0.596	1.020	0.184
forestFires	0.901	0.748	0.830	0.490	0.791	0.878	0.151
stock	0.129	0.176	1.361	0.753	0.225	1.746	0.364
steel	0.364	0.410	1.127	0.657	0.502	1.381	0.193
laser	0.274	0.330	1.205	0.484	0.365	1.334	0.152
concrete	0.576	0.659	1.145	0.622	0.784	1.361	0.137
treasury	0.079	0.120	1.523	0.541	0.165	2.093	0.180
mortgage	0.065	0.099	1.524	0.749	0.152	2.332	0.306
friedman	0.375	0.429	1.144	0.700	0.535	1.425	0.192
wizmir	0.199	0.215	1.081	0.481	0.274	1.377	0.140
wankara	0.185	0.206	1.115	0.673	0.297	1.604	0.139
plastic	0.463	0.452	0.975	0.551	0.512	1.107	0.223
quake	1.024	1.009	0.985	0.401	1.036	1.011	0.123
anacalt	0.242	0.399	1.652	0.504	0.478	1.978	0.233
abalone	0.713	0.738	1.035	0.444	0.780	1.094	0.166
delta-ail	0.582	0.585	1.004	0.475	0.630	1.081	0.180
puma32h	0.910	0.913	1.003	0.634	0.960	1.055	0.151
compactiv	0.281	0.303	1.079	0.447	0.387	1.379	0.105
delta-elv	0.626	0.639	1.020	0.556	0.670	1.070	0.150
tic	1.011	0.961	0.951	0.524	1.002	0.991	0.125
ailerons	0.522	0.528	1.011	0.437	0.581	1.112	0.153
pole	0.244	0.307	1.258	0.615	0.325	1.332	0.135
elevators	0.586	0.578	0.985	0.723	0.660	1.126	0.099
california	0.548	0.553	1.009	0.577	0.568	1.037	0.124
house	0.712	0.746	1.048	0.699	0.781	1.097	0.304
mv	0.160	0.205	1.279	0.578	0.221	1.380	0.494
average	0.476	0.496	1.116	0.557	0.555	1.307	0.185

**Table A10:** Experimental results for MEISR with 10-CV, IEA: *k*-NN (RIS), FPA: *k*-NN (RIS).

Dataset	r0	r1	r1/r0	c1	r2	r2/r0	c2
mach. CPU	0.392	0.407	1.038	0.385	0.426	1.086	0.216
baseball	0.606	0.623	1.029	0.591	0.753	1.244	0.110
dee	0.424	0.430	1.015	0.403	0.501	1.182	0.137
autoMPG8	0.364	0.403	1.108	0.499	0.480	1.320	0.160
autoMPG6	0.366	0.396	1.082	0.401	0.457	1.248	0.156
ele-1	0.584	0.562	0.963	0.489	0.575	0.985	0.178
forestFires	0.864	0.729	0.844	0.456	0.732	0.847	0.126
stock	0.105	0.140	1.338	0.689	0.181	1.729	0.371
steel	0.323	0.359	1.110	0.665	0.431	1.333	0.165
laser	0.204	0.243	1.193	0.489	0.271	1.330	0.128
concrete	0.521	0.593	1.138	0.593	0.678	1.303	0.129
treasury	0.058	0.088	1.503	0.531	0.118	2.015	0.159
mortgage	0.049	0.076	1.554	0.682	0.109	2.246	0.263
friedman	0.340	0.385	1.132	0.668	0.489	1.437	0.176
wizmir	0.178	0.192	1.081	0.491	0.238	1.339	0.117
wankara	0.167	0.183	1.096	0.643	0.273	1.635	0.127
plastic	0.468	0.451	0.965	0.504	0.520	1.112	0.184
quake	1.025	1.000	0.976	0.374	1.003	0.979	0.117
anacalt	0.212	0.352	1.661	0.510	0.404	1.904	0.226
abalone	0.690	0.709	1.028	0.435	0.730	1.059	0.136
delta-ail	0.560	0.566	1.011	0.477	0.580	1.035	0.170
puma32h	0.896	0.902	1.008	0.619	0.927	1.035	0.135
compactiv	0.240	0.259	1.077	0.441	0.333	1.388	0.097
delta-elv	0.610	0.610	1.000	0.546	0.627	1.027	0.123
tic	1.015	0.985	0.970	0.496	0.992	0.977	0.121
ailerons	0.504	0.511	1.015	0.421	0.538	1.069	0.128
pole	0.214	0.267	1.249	0.583	0.282	1.319	0.108
elevators	0.559	0.553	0.990	0.662	0.620	1.110	0.081
california	0.527	0.531	1.008	0.532	0.555	1.053	0.112
house	0.687	0.718	1.046	0.671	0.743	1.083	0.236
mv	0.140	0.179	1.280	0.540	0.186	1.328	0.420
average	0.448	0.465	1.113	0.532	0.508	1.282	0.165

**Table A11:** Relative $RMSE_{tst}$ for retention c1=0.5 and c2=0.25 (compression = 50% and 75%) in 50% holdout (average over 10 runs) for Threshold-Ensemble-CNN (TE-C), Threshold-Ensemble-ENN (TE-E), Discretization-Ensemble-CNN (DE-C), Discretization-Ensemble-ENN (DE-E) and MEISR. Inner evaluation algorithm *k*-NN with optimal *k* and binary instance selection. Final prediction algorithm: *k*-NN with optimal *k*.

	c1 = 0.50					c2 = 0.25				
Dataset	TE-C	TE-E	DE-C	DE-E	MEISR	TE-C	TE-E	DE-C	DE-E	MEISR
mach. CPU	1.270	1.065	1.362	1.022	1.149	1.537	1.296	1.755	1.393	1.202
baseball	1.118	1.087	1.155	1.266	1.035	1.346	1.310	1.734	1.598	1.183
dee	1.151	1.096	1.077	1.066	0.962	1.348	1.265	1.217	1.374	1.203
autoMPG8	1.255	1.086	1.185	1.121	0.976	1.538	1.308	1.280	1.248	1.198
autoMPG6	1.170	1.065	1.102	1.092	0.968	1.377	1.309	1.231	1.210	1.112
ele-1	1.047	1.058	1.028	1.066	0.962	1.080	1.179	1.075	1.160	1.014
stock	1.584	1.323	1.445	1.645	1.147	1.875	2.497	1.789	2.249	1.433
laser	1.466	1.142	1.543	1.132	1.038	1.520	1.606	1.797	1.282	1.184
concrete	1.279	1.156	1.225	1.136	1.096	1.382	1.278	1.338	1.365	1.193
treasury	1.544	1.589	1.694	1.351	1.093	2.072	3.062	2.360	3.260	1.990
mortgage	1.426	1.568	1.799	1.305	1.281	2.049	4.032	2.476	3.241	2.101
friedman	1.213	1.199	1.147	1.173	1.092	1.376	1.420	1.316	1.415	1.305
wizmir	1.289	1.224	1.227	1.146	1.081	1.365	1.389	1.356	1.371	1.131
wankara	1.214	1.220	1.145	1.151	1.076	1.422	1.588	1.401	1.489	1.211
plastic	1.025	1.069	1.033	1.523	0.994	1.033	1.155	1.285	1.957	0.989
quake	1.238	1.023	1.126	1.064	0.992	1.456	1.055	1.187	1.133	1.005
abalone	1.045	1.012	1.137	1.074	1.023	1.093	1.039	1.210	1.148	1.011
compactiv	1.129	1.274	1.277	1.366	1.125	1.302	2.862	1.328	1.428	1.200
tic	1.236	1.013	1.069	1.003	0.992	1.429	1.046	1.286	0.996	0.996
ailerons	1.126	1.033	1.198	1.048	1.028	1.238	1.126	1.236	1.172	1.088
pole	1.351	1.066	1.420	1.049	1.117	1.501	1.747	1.598	1.796	1.234
elevators	1.126	1.059	1.236	1.052	1.041	1.202	1.190	1.291	1.157	1.097
california	1.103	1.115	1.077	1.080	1.037	1.259	1.216	1.197	1.146	1.234
house	1.073	1.093	1.253	1.062	1.036	1.222	1.146	1.304	1.258	1.073
average	1.228	1.152	1.248	1.166	1.056	1.418	1.588	1.460	1.535	1.224
times best	2	0	0	1	21	2	0	0	0	22
*t*-test *p*	0.0000	0.005	0.0000	0.0022		0.0085	0.0158	0.0070	0.0128	
Wilcoxon *p*	0.0000	0.0000	0.0000	0.0000		0.0001	0.0000	0.0000	0.0000	

**Table A12:** Relative $RMSE_{tst}$ for retention c1=0.5 and c2=0.25 in 10-fold cross-validation with *k*-NN with optimal *k*. All symbols are explained in the caption of Table A11.

	c1 = 0.50					c2 = 0.25				
Dataset	TE-C	TE-E	DE-C	DE-E	MEISR	TE-C	TE-E	DE-C	DE-E	MEISR
mach. CPU	1.267	1.048	1.360	1.013	1.052	1.373	1.227	1.547	1.253	1.148
baseball	1.081	1.062	1.171	1.235	0.997	1.183	1.149	1.629	1.406	1.094
dee	1.129	1.105	1.058	1.058	1.007	1.199	1.199	1.110	1.293	1.031
autoMPG8	1.226	1.098	1.172	1.120	1.031	1.362	1.226	1.199	1.199	1.138
autoMPG6	1.127	1.082	1.071	1.088	1.043	1.280	1.197	1.141	1.197	1.074
ele-1	1.019	1.029	1.041	1.039	0.954	1.056	1.100	1.054	1.097	1.048
stock	1.549	1.345	1.442	1.646	1.180	1.849	2.401	1.771	2.236	1.551
laser	1.409	1.155	1.570	1.118	1.031	1.501	1.501	1.723	1.293	1.232
concrete	1.240	1.181	1.192	1.111	1.054	1.346	1.308	1.346	1.308	1.224
treasury	1.491	1.563	1.763	1.327	1.302	1.982	3.091	2.364	3.273	1.372
mortgage	1.374	1.555	1.858	1.273	1.304	2.020	4.081	2.404	3.111	2.854
friedman	1.199	1.214	1.191	1.156	1.073	1.313	1.410	1.299	1.343	1.234
wizmir	1.243	1.200	1.217	1.122	1.060	1.362	1.362	1.362	1.362	1.264
wankara	1.212	1.212	1.164	1.146	1.098	1.373	1.492	1.373	1.403	1.254
plastic	0.991	1.071	1.006	1.519	0.969	1.022	1.138	1.253	1.909	0.989
quake	1.235	1.012	1.102	1.056	0.990	1.455	1.015	1.144	1.102	0.998
abalone	1.026	1.026	1.121	1.079	1.031	1.036	1.055	1.166	1.103	1.058
compactiv	1.107	1.293	1.281	1.341	1.209	1.245	2.810	1.331	1.348	1.241
tic	1.221	1.001	1.113	1.000	0.986	1.388	1.002	1.262	1.001	0.993
ailerons	1.096	1.042	1.158	1.054	1.031	1.170	1.102	1.251	1.138	1.096
pole	1.339	1.082	1.478	1.022	1.125	1.483	1.684	1.604	1.698	1.261
elevators	1.092	1.050	1.223	1.040	1.039	1.129	1.109	1.310	1.129	1.103
california	1.082	1.094	1.117	1.077	1.037	1.204	1.147	1.177	1.154	1.116
house	1.075	1.081	1.208	1.034	1.029	1.159	1.085	1.250	1.250	1.078
average	1.201	1.148	1.253	1.153	1.068	1.354	1.537	1.420	1.484	1.227
times best	1	1	0	3	19	1	0	0	1	22
*t*-test *p*	0.0000	0.0000	0.0000	0.0000		0.0235	0.0059	0.0003	0.0167	
Wilcoxon *p*	0.0001	0.0001	0.0000	0.0008		0.0004	0.0000	0.0000	0.0004	

**Table A13:** Relative $1-r^{2}$ in 50% houdout for DROP3-RT and MEISR for retention c1=0.5 and c2=0.25 with 8-NN. Dr2: relative $1-r^{2}$ with DROP3-RT. Mr2: relative $1-r^{2}$ with MEISR, Dc1: retention rate with DROP3-RT, Mc1: retention rate with MEISR, $r^{2}$ is the correlation between the predicted and actual output.

Dataset	Dr2	Mr2	Mr2/Dr2	Dc1	Mc1	Mc1/Dc1
mach. CPU	1.461	1.242	0.850	0.505	0.396	0.784
baseball	1.205	1.182	0.980	0.472	0.341	0.723
dee	1.120	1.074	0.959	0.523	0.315	0.602
autoMPG8	1.211	1.116	0.921	0.539	0.387	0.718
autoMPG6	1.109	1.171	1.056	0.536	0.345	0.644
ele-1	1.082	0.847	0.783	0.530	0.314	0.593
stock	1.420	1.503	1.058	0.583	0.432	0.742
laser	1.381	1.017	0.736	0.659	0.443	0.673
concrete	1.292	1.224	0.948	0.507	0.410	0.808
treasury	1.419	1.470	1.036	0.636	0.421	0.662
friedman	1.195	1.153	0.964	0.574	0.434	0.756
wizmir	1.260	1.101	0.873	0.569	0.324	0.569
wankara	1.135	1.188	1.047	0.571	0.382	0.669
plastic	1.050	0.797	0.759	0.391	0.378	0.967
quake	1.078	0.962	0.892	0.438	0.403	0.920
abalone	1.031	1.081	1.049	0.455	0.432	0.949
$delta_{a}il$	1.040	1.037	0.997	0.445	0.425	0.955
puma32h	1.642	1.537	0.935	0.418	0.402	0.963
compactiv	1.023	1.094	1.070	0.485	0.429	0.885
$delta_{e}lv$	1.051	1.043	0.992	0.469	0.438	0.933
ailerons	1.447	1.049	0.725	0.457	0.417	0.912
pole	1.151	1.139	0.990	0.335	0.409	1.222
elevators	1.066	1.150	1.079	0.470	0.416	0.885
california	1.089	1.072	0.984	0.502	0.474	0.944
house	1.136	0.974	0.858	0.457	0.428	0.938
mv	1.203	1.300	1.080	0.609	0.530	0.870
average	1.204	1.135	0.935	0.505	0.405	0.819
times best	8	18		1	25	
*t*-test *p*			0.0773			0.0000
Wilcoxon *p*			0.0566			0.0000

**Table A14:** Relative $1-r^{2}$ in 10-fold cross-validation for DROP3-RT and MEISR for retention c1=0.5 and c2=0.25 with 8-NN. All symbols are explained in the caption of Table A13.

Dataset	Dr2	Mr2	Mr2/Dr2	Dc1	Mc1	Mc1/Dc1
mach. CPU	1.541	1.263	0.819	0.495	0.374	0.756
baseball	1.160	1.134	0.978	0.460	0.395	0.859
dee	1.166	0.991	0.850	0.511	0.352	0.689
autoMPG8	1.210	1.118	0.924	0.491	0.421	0.857
autoMPG6	1.168	1.137	0.974	0.511	0.373	0.729
ele-1	1.023	0.892	0.873	0.486	0.353	0.725
stock	1.685	1.421	0.843	0.572	0.483	0.843
laser	1.436	1.079	0.751	0.605	0.455	0.752
concrete	1.351	1.235	0.914	0.502	0.465	0.926
treasury	1.346	1.500	1.114	0.620	0.439	0.707
friedman	1.076	1.186	1.102	0.538	0.437	0.812
wizmir	1.329	1.140	0.858	0.510	0.410	0.803
wankara	1.355	1.103	0.814	0.521	0.444	0.853
plastic	0.964	0.833	0.864	0.419	0.390	0.930
quake	1.058	1.044	0.986	0.420	0.387	0.922
abalone	1.053	1.034	0.982	0.416	0.389	0.935
delta-ail	1.039	1.001	0.963	0.433	0.364	0.841
puma32h	1.032	1.062	1.028	0.381	0.375	0.986
compactiv	1.557	1.066	0.685	0.449	0.391	0.870
delta-elv	1.016	1.011	0.995	0.432	0.376	0.870
ailerons	1.039	1.134	1.092	0.425	0.264	0.620
pole	1.425	1.696	1.190	0.245	0.244	0.996
elevators	1.137	1.135	0.998	0.438	0.358	0.816
california	1.048	1.105	1.055	0.475	0.402	0.845
house	1.084	1.041	0.969	0.430	0.318	0.741
mv	1.061	1.297	1.222	0.531	0.392	0.739
average	1.228	1.161	0.953	0.481	0.391	0.822
times best	8	19		0	26	
*t*-test *p*			0.1112			0.0001
Wilcoxon *p*			0.0588			0.0001

**Table A15:** Statistical significance test for experiments presented in Table A3, Table A4, Table A5, Table A6, Table A7, Table A8, Table A9 and Table A10.

Algorithm	Relation	*t*-test *p*	Wilco-xon *p*	relation	*t*-test *p*	Wilco-xon *p*
MEISR, IEA: 1-NN (BIS), FPA: 1-NN (BIS), 50%H	r1/r0	0.0148	0.0026	r2/r0	0.4708	0.0018
MEISR, IEA: 1-NN (BIS), FPA: 1-NN (BIS), 10-CV	r1/r0	0.0148	0.0026	r2/r0	0.4708	0.0018
MEISR, IEA: *k*-NN (BIS), FPA: *k*-NN (BIS), 50%H	r1/r0	0.0259	0.0182	r2/r0	0.0001	0.0001
MEISR, IEA: *k*-NN (BIS), FPA: *k*-NN (BIS), 10-CV	r1/r0	0.2309	0.0058	r2/r0	0.0001	0.0001
MEISR, IEA: *k*-NN (BIS), FPA: MLP (BIS), 50%H	r1/r0	0.5664	0.4839	r2/r0	0.0001	0.0001
MEISR, IEA: *k*-NN (BIS), FPA: MLP (BIS), 10-CV	r1/r0	0.4782	0.4593	r2/r0	0.0003	0.0001
MEISR, IEA: *k*-NN (RIS), FPA: *k*-NN (RIS), 50%H	r1/r0	0.0305	0.0022	r2/r0	0.0001	0.0001
MEISR, IEA: *k*-NN (RIS), FPA: *k*-NN (RIS), 10-CV	r1/r0	0.0393	0.0032	r2/r0	0.0001	0.0001

**Table A16:** Calculation time of the MEISR algorithm for BIS using our software (available from the online supplementary resources) on a two Xeon X5-2696v2 machine. inst—number of instances, at—number of attributes, dist, 1−NNt and kNNt—total time of the process, where the inner regressor was respectively 1-NN and *k*-NN with optimal *k*, 1d% and kd%—percentage of total time used for calculating the distance matrix with respectively 1-NN and *k*-NN with optimal *k* as inner regressors, 1−NNti and kNNti—total process time per one instance.

Dataset	inst	at	k	1-NNt [s]	kNNt [s]	dist [s]	1d% [%]	kd% [%]	1-NNti [ms]	kNNti [ms]
mach. CPU	188	6	1	0.119	0.119	0.011	9.3	9.3	0.633	0.633
baseball	303	16	7	0.179	0.215	0.018	9.8	8.1	0.590	0.709
dee	329	6	7	0.181	0.219	0.014	7.8	6.5	0.551	0.667
autoMPG8	353	7	6	0.185	0.206	0.016	8.6	7.7	0.524	0.584
autoMPG6	353	5	4	0.186	0.202	0.015	8.0	7.3	0.527	0.573
ele-1	446	2	11	0.243	0.319	0.017	6.8	5.2	0.545	0.716
forestFire	465	12	11	0.293	0.343	0.024	8.1	6.9	0.630	0.737
stock	855	9	3	0.474	0.491	0.043	9.1	8.8	0.554	0.574
steel	864	12	4	0.462	0.481	0.046	9.8	9.5	0.535	0.557
laser	894	4	3	0.481	0.483	0.036	7.6	7.5	0.538	0.540
concrete	927	8	4	0.494	0.536	0.047	9.5	8.8	0.533	0.578
treasury	944	15	3	0.491	0.506	0.066	13.5	13.1	0.520	0.536
mortgage	944	15	2	0.508	0.513	0.058	11.4	11.3	0.538	0.543
friedman	1080	5	7	0.548	0.612	0.048	8.7	7.8	0.507	0.567
wizmir	1315	9	7	0.647	0.731	0.077	11.8	10.5	0.492	0.556
wankara	1448	9	9	0.671	0.802	0.080	11.9	10.0	0.463	0.554
plastic	1485	2	11	0.659	0.905	0.065	9.9	7.2	0.444	0.609
quake	1960	3	11	0.851	1.070	0.123	14.5	11.5	0.434	0.546
anacalt	3647	7	2	1.58	1.61	0.309	19.6	19.2	0.433	0.441
abalone	3759	8	11	1.68	2.25	0.341	20.3	15.2	0.447	0.599
delta-ail	6415	5	11	2.93	3.31	0.749	25.6	22.6	0.457	0.516
puma32h	7372	32	11	4.09	5.06	1.67	40.8	33.0	0.555	0.686
compactiv	7373	21	2	3.91	3.93	1.45	37.1	36.9	0.530	0.533
delta-elv	8564	6	11	4.11	5.19	2.06	50.1	39.7	0.480	0.606
tic	8840	85	11	6.41	7.81	3.93	61.3	50.3	0.725	0.884
ailerons	12375	40	10	8.61	10.1	4.88	56.7	48.3	0.696	0.816
pole	13498	26	4	8.52	10.3	4.97	58.3	48.3	0.631	0.763
elevators	14938	18	8	10.4	12.7	5.13	49.3	44.7	0.696	0.850
california	18576	8	9	16.0	18.2	9.27	57.9	50.9	0.861	0.980
house	20506	16	11	18.6	21.1	11.3	60.8	53.6	0.907	1.029
part of mv	27178	10	9	28.1	30.3	17.7	63.0	58.4	1.034	1.115
mv	36690	10	9	47.8	50.0	32.1	67.1	64.2	1.303	1.363
